# Dissociable effects of game elements on motivation and cognition in a task-switching training in middle childhood

**DOI:** 10.3389/fpsyg.2014.01275

**Published:** 2014-11-13

**Authors:** Sandra Dörrenbächer, Philipp M. Müller, Johannes Tröger, Jutta Kray

**Affiliations:** Department of Psychology, Development of Language, Learning and Action, Saarland UniversitySaarbrücken, Germany

**Keywords:** cognitive control, task switching, motivation, video-game elements, training, transfer, middle childhood

## Abstract

Although motivational reinforcers are often used to enhance the attractiveness of trainings of cognitive control in children, little is known about how such motivational manipulations of the setting contribute to separate gains in motivation and cognitive-control performance. Here we provide a framework for systematically investigating the impact of a motivational video-game setting on the training motivation, the task performance, and the transfer success in a task-switching training in middle-aged children (8–11 years of age). We manipulated both the type of training (low-demanding/single-task training vs. high-demanding/task-switching training) as well as the motivational setting (low-motivational/without video-game elements vs. high-motivational/with video-game elements) separately from another. The results indicated that the addition of game elements to a training setting enhanced the intrinsic interest in task practice, independently of the cognitive demands placed by the training type. In the task-switching group, the high-motivational training setting led to an additional enhancement of task and switching performance during the training phase right from the outset. These motivation-induced benefits projected onto the switching performance in a switching situation different from the trained one (near-transfer measurement). However, in structurally dissimilar cognitive tasks (far-transfer measurement), the motivational gains only transferred to the response dynamics (speed of processing). Hence, the motivational setting clearly had a positive impact on the training motivation and on the paradigm-specific task-switching abilities; it did not, however, consistently generalize on broad cognitive processes. These findings shed new light on the conflation of motivation and cognition in childhood and may help to refine guidelines for designing adequate training interventions.

## Introduction

### Cognitive control in middle childhood

Human thinking and acting require a high amount of flexibility to cope with fast-moving environmental challenges of everyday life. Especially in late middle childhood or in preadolescence (i.e., between the approximate ages of 8 and 11 years), there seems to be a disproportionate ratio of cognitive and behavioral adaptability (Eccles, 1999; Mizuno et al., 2011): While children's developmental status has not fully matured yet, there is nevertheless a growing number of changeable task demands that call for an autonomous alignment along internal and external standards or goals. The ability to react flexibly to such changing environmental conditions seems to be strongly mediated by the maturation of cognitive control throughout middle childhood (Miyake et al., 2000; Monsell, 2003; Barkley, 2012). Cognitive control or executive functioning is a fundamental aspect of human intelligence and encompasses a set of interrelated mental processes enabling people to guide thoughts and actions according to their external and internal goals (e.g., Kray and Ferdinand, 2013). The processes that are involved in this cluster are very elusive in widespread psychological research (e.g., Barkley, 2012). However, there is growing consensus on some putative core components, namely working memory (WM), inhibition, and mental flexibility (for recent reviews see Diamond, 2012; Kray and Ferdinand, 2013). These components are separable but also interrelated (see also Miyake et al., 2000).

The efficiency of cognitive-control abilities is closely related to highly important skills for children's everyday flexibility needs, such as academic activities or social appropriateness. Cognitive control is assumed to predict reading or arithmetic abilities (Van der Sluis et al., 2007; Clark et al., 2010), to refer to cross-curricular skills, such as self-regulated learning (Garner, 2009), or to lead to the support of good habits and adequate classroom-behavior (Riggs et al., 2003). Thus, the enhancement of cognitive control may be particularly important in middle childhood as it may lead to a synergy with developmental tasks. For the purpose of the present study, we specifically selected children in this preadolescent age range and measured the effectiveness of a training intervention aimed at improving cognitive control. The important new insight we hope to gain is how motivational variables influence the effectiveness of this training. Specifically, the focus of the present study is to examine the potential reinforcement by the training setting (here by adding game elements in order to enhance the training effort).

The effectiveness of a training intervention can be measured by direct training benefits and, more importantly, by indirect transfer effects (e.g., Karbach and Kray, 2009). Training benefits refer to performance improvements in a specifically trained task, while transfer refers to indirect training gains in task domains different from the trained ones (ibid.). Here, we will distinguish between near and far transfer effects. By near transfer, we mean the generalization of training-induced improvements to a new but structurally similar task, while far transfer refers to a broader generalization of training-induced improvements to dissimilar task domains or theoretical constructs (Karbach, 2008; Klingberg, 2010). The present study investigates how a motivational training setting modulates such training and transfer effects on flexible behavioral control.

One approach, which examines this flexible control, is the task-switching paradigm (for a review, see Kiesel et al., 2010). In this paradigm, participants are instructed to perform two simple categorization tasks (referred to as “A” and “B”) while the stimuli to be classified are often bivalent, that is, they contain properties that are likewise relevant for both tasks (Cragg and Chevalier, 2012). The tasks are implemented in two types of blocks: In single-task blocks, participants have to perform either task A *or* B separately from each other, while in mixed-task blocks, they have to switch between both tasks A *and* B within the same block. The sequence for switching between tasks A and B within mixed blocks can either be determined by external cues, explicitly indicating the next task from trial to trial (cued variant of the task-switching paradigm, e.g., Crone et al., 2004); or the sequence can be prespecified, which means that the participants have to switch the task on every second trial (alternating-runs variant of the task-switching paradigm, e.g., Karbach and Kray, 2009; for a review, see Cragg and Chevalier, 2012). The latter variant requires the participants to keep track of the task sequence without external memory aid. We will further rely on the alternating-runs paradigm, which imposes a higher level of cognitive demands on internal updating.

The advantage of the task-switching paradigm is that it allows for a common measurement as well as a separation of the different components of cognitive control by determining different types of costs (Kiesel et al., 2010): Mixing costs are defined as the difference in mean performance between single-task and mixed-task blocks. They refer to the ability to maintain and to select two task sets (Kray and Lindenberger, 2000). Switching costs are defined as the difference in mean performance between non-switch trials and switch trials within mixed-task blocks. They are associated with the task shift *per se*. Besides, the ambiguous stimuli continuously put high demands on the participant's interference control.

So far, only a few studies have applied the task-switching paradigm in middle-aged children. However, these reported substantial improvements in cognitive-control components by practice in switching (e.g., Karbach and Kray, 2009; Kray et al., 2012). For instance, Karbach and Kray (2009) have investigated the effectiveness of a task-switching training in middle-aged children (age range: 8–10 years) within the broader framework of a lifespan study. They used a pretest-training-posttest design with numerous treatment groups and an active control group. The treatment groups were all trained in task switching, i.e., in mixed-task blocks requiring high demands on cognitive control. The active control group performed identical tasks but was trained in single-task blocks requiring low demands on cognitive control. The results of this study revealed larger near transfer effects of the task-switching training in children compared to younger adults than the active control group. The results also showed far transfer effects to inhibition as well as verbal and visuospatial WM or fluid intelligence. Comparable performance gains from a task-switching training on inhibition and verbal WM were also found for children with attention deficit-/hyperactivity disorder (ADHD; age range: 7–12 years; Kray et al., 2012). It is notable that Karbach and Kray (2009) as well as Kray et al. (2012) used an identical training setting for all age groups. In each study, the researchers did not apply a more child-friendly version of the task-switching training, for example by adding game elements or cover stories. This resulted in a relatively high drop-out rate, at least in the ADHD study. Thus, we had reasonable grounds for the present study to refine guidelines for a task-switching training in middle childhood by increasing the motivational incitement of the training setting.

Most of the recent training studies that examined the plasticity of cognitive control in childhood have specifically focused on the modifiability of working-memory abilities (for a meta-analysis on the effectiveness of working-memory trainings, see Melby-Lervåg and Hulme, 2013). Although a direct training benefit of practicing WM tasks has indeed widely been supported, there is, however, no consensus on the limitations of the transfer's scope yet (e.g., Klingberg, 2010; Diamond and Lee, 2011). Some studies point to strong transfer effects, both in untrained but structurally similar WM tasks (near transfer) and in other cognitive domains, such as interference control or even reasoning (far transfer, e.g., Klingberg et al., 2002, 2005). Yet, other studies either found a much smaller scope of transfer benefits or they yielded quite mixed findings by little transfer to other cognitive domains and substantial transfer to academic achievement (e.g., Holmes et al., 2009; Egeland et al., 2013; Karbach et al., 2014). Trainings that aimed at improving inhibition in children have been shown to be less promising than WM trainings (e.g., Thorell et al., 2009) although it should be noted that few studies have applied a specific inhibition training. In the present study, we used a task-switching training designed to tap into several executive-control processes at once with the aim of broadening the cognitive transfer.

Some of the available laboratory or commercialized cognitive trainings for children already use game elements, cover stories, and specific types of incentive presentation in order to enhance and maintain training motivation throughout the intervention (e.g., Klingberg et al., 2005; Prins et al., 2011; Bioulac et al., 2014). However, so far it is not quite clear how and whether such modifications of the training setting indeed influence training motivation and training success. Thus, variations in the training setting across studies may also contribute to these mixed outcomes of previous training interventions in children. In the following, we will therefore briefly introduce how motivational variables, and in particular the training setting enriched by game elements, are considered to influence cognitive control.

### Motivational influences on cognitive control

Motivation can generally be defined as a current sensational state that modulates the (cognitive or behavioral) effort an organism is willing to invest to achieve internal or external goals (Locke and Braver, 2010). As motivation aligns goal-directed behavior, it is closely related to cognitive control (see also the evidence from cognitive neuroscience, Locke and Braver, 2008, 2010; Kouneiher et al., 2009) and may particularly relate to task switching (Kleinsorge and Rinkenauer, 2012). Developmental researchers like Zelazo et al. (2010) propose an interplay between *cold* cognitive and *hot* affective-motivational processes apparent during cognitive-control engagement whereas, due to a differential maturation of these processes, hot influences seem to be especially pronounced in childhood. Zelazo et al. (2010) suggest that certain hot contexts may facilitate executive functioning, and especially cognitive flexibility, which is considered to be at the core of cognitive control. Therefore, the stimulation of one's motivational state by “heating up” the context to a certain degree will maybe enhance the engagement invested in meeting the cognitive-control task demands (ibid.).

However, similar to the construct of cognitive control, motivation is multi-faceted in nature (Reiss, 2004) and lacks both conceptual and operational clarity. There is a variety of personal motivational tendencies considered to be relatively invariant across situations, such as achievement goal orientations (e.g., Meece et al., 2006), self-efficacy beliefs (e.g., Bandura, 1997), the need for cognition (e.g., Cacioppo et al., 1996), interests (e.g., Krapp, 2002), temperament dimensions (e.g., Colom et al., 2007), or habitual patterns of causal outcome interpretations (i.e., attributional styles, e.g., Russell, 1982). Current motivation certainly depends on such dispositional differences. Nonetheless, specific motivated behavior is only expressed in interaction with current situational or environmental properties. This environmental input may, in turn, take various forms of reinforcement contingencies. Current motivation thus arises from and becomes apparent in a complex interplay between various personal and environmental determinants.

For the purpose of the present study, we mainly rely on the concept of intrinsic interest that directly links environment to personality variables. The self-determination theory (SDT; Ryan and Deci, 2000) defines the intrinsic interest as the inherent satisfaction obtained from a learning task or action, which has become internalized as a personal value. That is to say, if *environmental* properties enhance the attractiveness of a task so that this task is being perceived as interesting *per se*, this interest will be incorporated into the *personal* self-identity. In the present study, we therefore intended to optimize the environmental features of the training setting in order to induce children's intrinsic interest in the training task. In sum, three basic psychological needs are assumed to trigger the intrinsic interest: (1) *Relatedness* refers to the need for social integration and responsibility; (2) *Autonomy* refers to the need for perceiving oneself as the origin of acting or as being independent from external influences; (3) *Competency* relates to the need for challenge and to feelings of efficacy (ibid.). The systematic manipulation of these three subcomponents in a training environment should result in the training task being “hot” or exciting, thus fostering children's training interest. This should also stimulate the interaction of this “heated” motivational state with “cold” cognitive-control performance (Zelazo et al., 2010). In order to promote intrinsic interest of children in the task-switching training, we created a video-game training setting (for reviews on video-game playing see also Green and Bavelier, 2006; Bavelier et al., 2012). In particular, adding game elements to the training setting would allow us to vary the training context right along the specific meaning of the SDT. Accordingly, Ryan et al. (2006) specify the motivational pull of video games in terms of these theorized needs for relatedness, autonomy, and competency: In the context of computerized games, the authors associate relatedness with presence, that is, the sense of being *within* the game or being part of the game. Game relatedness is thus rewritten as a feeling of medial integration and social responsiveness to the collective of game characters. Autonomy in game environments refers to provisions of choice, informational feedback by means of rewards, and non-controlling instructions. Competency is related to the need for challenge in terms of opportunities to acquire new abilities as well as skills for playing the game successfully.

A growing body of research recognizes the great value of these video-game implementations, even for cognitive trainings in children (e.g., Klingberg et al., 2005; Prins et al., 2011, 2013; Dovis et al., 2012; Van der Oord et al., 2012). Much of this research, however, does not directly test the beneficial effect of the game setting on intrinsic interest and task effort. In the present study, we therefore systematically examine the impact of the addition of game elements to the training setting in order to determine how motivation first affects the training situation and second promotes the transfer of training.

### A game-based framework for a cognitive-control training in children

**Figure 1:**
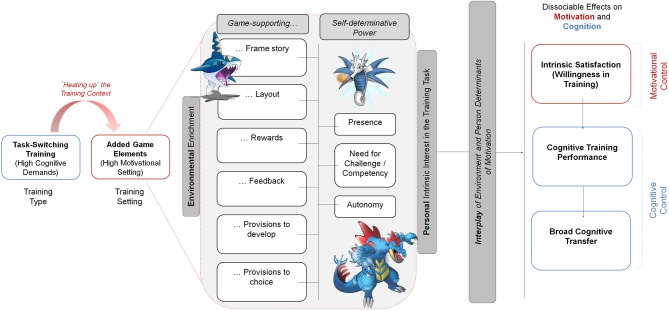
**Depiction of our proposed game-based framework for a task-switching training in middle childhood**. The context (setting) of the cognitive-control training should be “heated up” by adding game elements. This environmental enrichment should induce self-determinative feelings, leading to the formation of a personal interest in the training task. The interplay of environmental enrichment and personal interest should, in turn, differentially affect the motivational and cognitive outcomes. Blue ink points to rather “cold” cognitive facets; red ink points to rather “hot” motivational facets (following the notion of Zelazo et al., 2010).

The primary aim of this study was to shed light on the impact of the motivational video-game setting on the task-switching training to examine the interaction between motivation and cognitive control. We created a training environment that should be perceived as intrinsically interesting, thereby inducing self-determinative feelings (Ryan and Deci, 2000). These should have a positive impact on the training willingness and should, as a consequence, result in better cognitive training performance (Zelazo et al., 2010) that may generalize to other cognitive tasks. Figure 1 summarizes the rationale of our hypothesized framework allowing for the interplay between cognitive control and motivation. To examine this interaction between cognition and motivation, we varied two experimental factors, namely the Training Type (low-demanding/single-task control vs. high-demanding/task-switching training) and the Training Setting (low-motivational/without adding game elements vs. high-motivational/with adding game elements). In order to maximize the motivational pull of the training setting, we manipulated the degree of the senses of presence, autonomy, and competency in the task environment that will be described in details in the following.

#### Story framework

In the low-motivational condition, the procedure and instructions were presented without any narrative background. In the high-motivational condition, however, the training was embedded in a frame story. The first training session started with a video showing the story of an astronaut who made a forced landing with his spaceship on a foreign planet, *Aquatek*. Participants were asked to adopt the role of this delineated astronaut: To be able to repair the damaged spaceship, participants had to train the planet's natives, the *Watermons*, and they had to fight Watermon battles to earn *Aquatek money*. Engagement in these imaginative Watermon battles corresponded to high performance on the training task. By creating this frame story and providing children with the possibility to identify with the protagonist, we assumed to foster the children's sense of being part of the game world as well as their experience of presence.

#### Stimulus material

**Figure 2:**
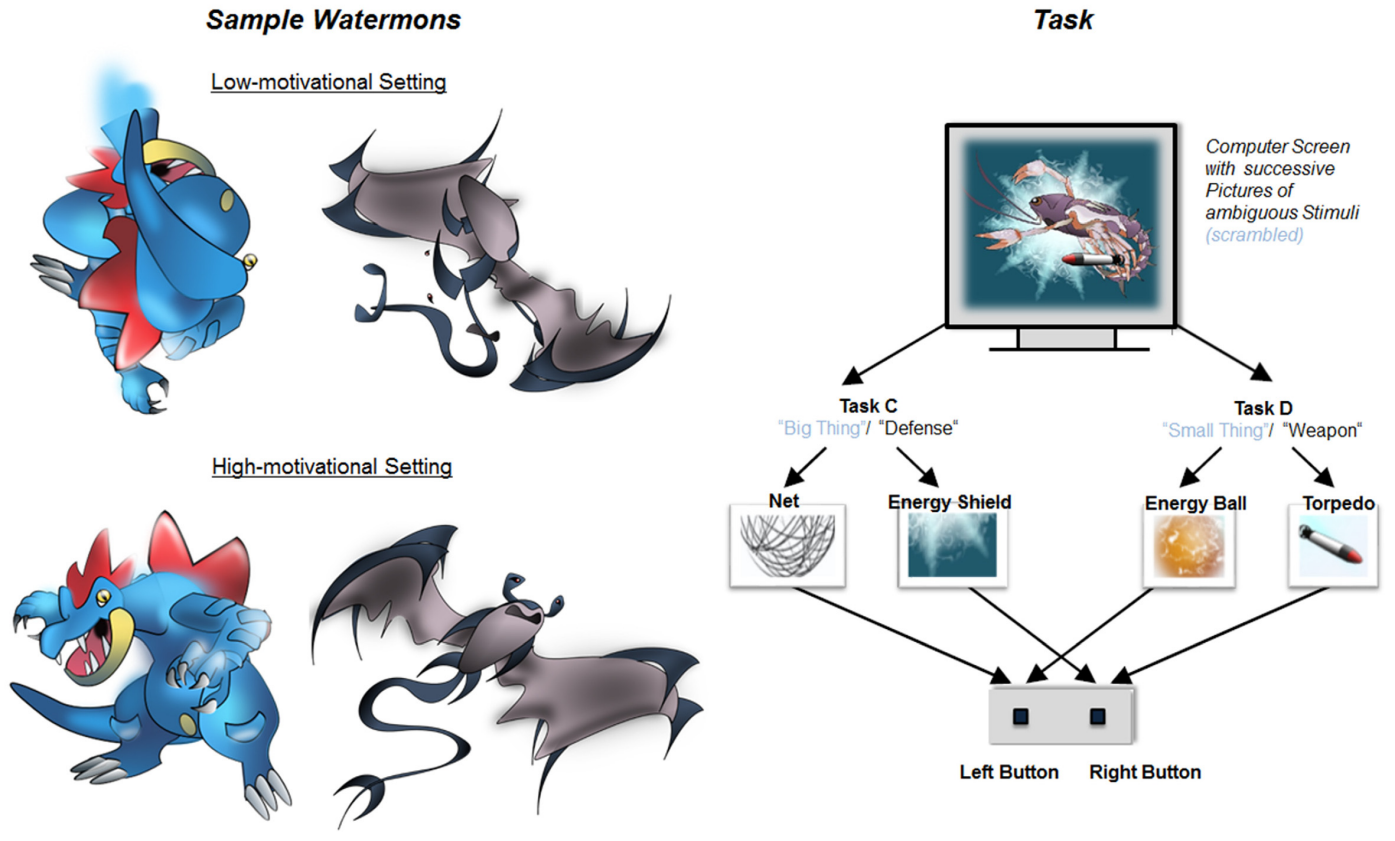
**Sample stimuli (left panel) and overview of the employed task-switching paradigm (right panel) for the low- and the high-motivational condition**. Discrepancies in the low-motivational group are highlighted in blue ink.

All groups (low- and high-motivational) were equally presented with pictures containing Watermons in order to keep the perceptual properties of the task-relevant stimuli constant across the groups. The Watermons were not explicitly labeled as such in the low-motivational condition. Moreover, the low-motivational groups saw a scrambled, de-contextualized version of the Watermons. This version lacked the framing sensation of animated characters (see Figure 2). In the high-motivational conditions, in contrast, the game relatedness and the feeling of social responsiveness to the collective of the Watermon characters was explicitly encouraged by employing context-tied stimuli.

#### Categorization tasks

All groups performed the same training tasks, however with a different labeling. In the low-motivational groups, children were instructed to perform a “big thing” task and a “small thing” task (see Figure 2). In the high-motivational groups, the two task labels fitted to the game context, that is, children were instructed to perform a “defense” task and a “weapon” task (for details, see Section Training intervention). By labeling the tasks in such a game-related manner, we intended to modulate the children's sense of being part of an authentic story.

#### Training-goal instructions

In the low-motivational groups, children were asked by means of a neutral text slide to make their decisions as accurately and as fast as possible. The high-motivational groups received the same instruction, but embedded in a more playful context including incentives which led to virtual rewards. During training, the players could gain experience points for each correct response. In this way, the fighting Watermon was charged up with energy until it went to the next stage of development, looking bigger and stronger. By giving children the opportunity to become more powerful, we aimed at nurturing the children's sense of competency or their need for challenge. In addition, children in the high-motivational condition could improve their “tournament status” by winning most task blocks. If the tournament status was positive at the end of a training session, players won this complete tournament and received a new Watermon for their collection. In the subsequent sessions, they then had the chance to select Watermons freely from this collection and send them into the next battle. By giving children this option, we intended to afford an autonomous game environment.

#### Feedback

**Figure 3:**
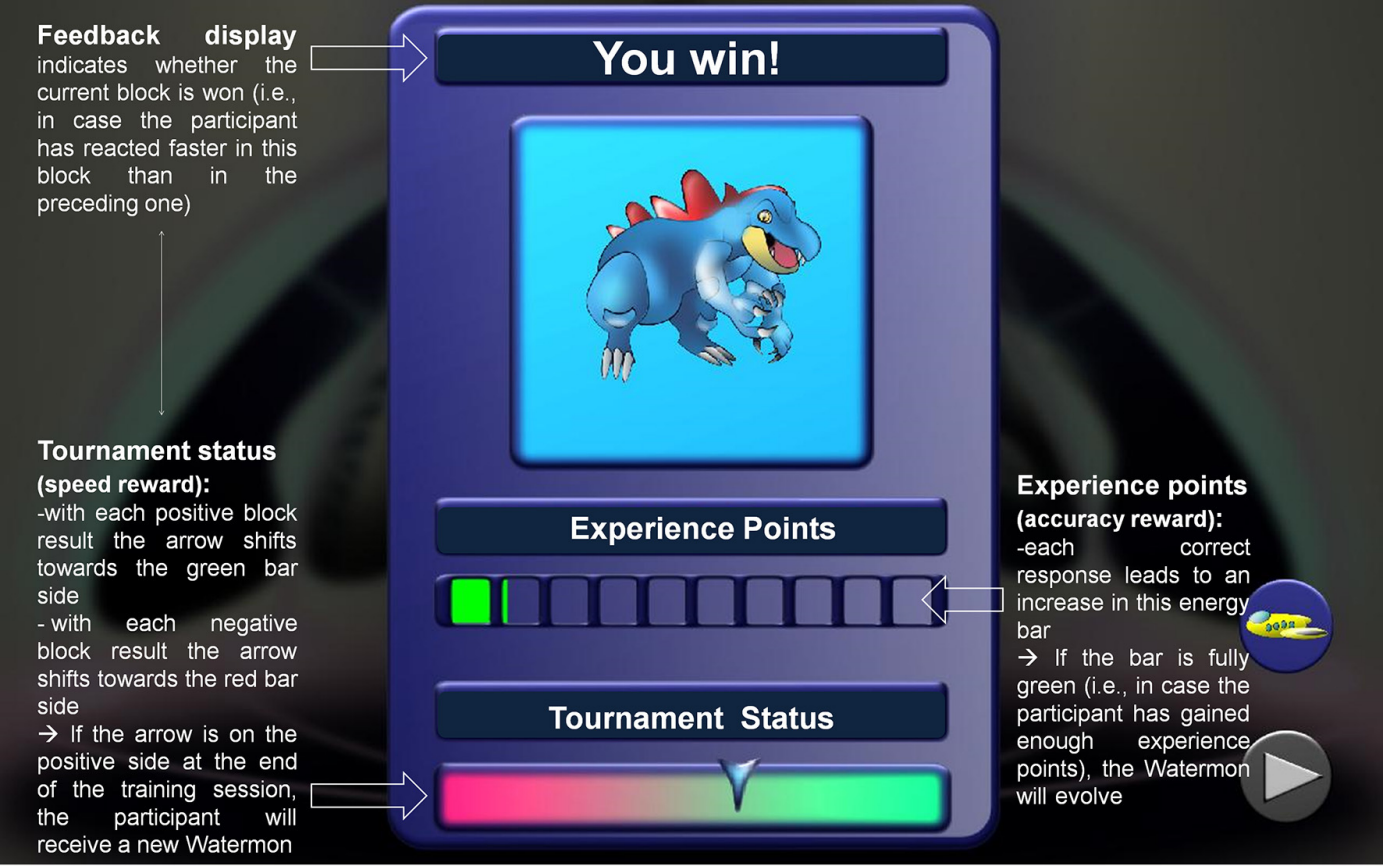
**Feedback display in the high-motivational condition**.

Participants in all training groups received adaptive feedback about their percentage of correct responses as well as response speed (for details, see Section Training intervention). In the low-motivational groups, this feedback was presented in a simple text slide. In contrast, in the high-motivational groups, feedback was presented in a game-supporting layout and should thus be experienced as highly encouraging: Children saw an illustrative overview card indicating their success or failure, their Watermon's experience points, and their current tournament status (see Figure 3). The use of rewards in the form of informational feedback should also raise the sense of autonomy and competency.

To sum up, the variations of the task-design elements were assumed to tap the basic needs of the self-determinative power of video-games. By inducing such self-determinative feelings, the interest in the training task should become internalized, leading to a robust formation of personal motivation. The interplay between environmental enrichment and personal interest should then nurture the training willingness, and by this, should lead to better cognitive training performance that may also generalize to other cognitive tasks.

### Empirical evidence for the effects of motivation on cognitive training interventions

Prins et al. (2011) addressed the question of motivational effects of a game environment on improvements in cognitive control. They trained two clinical subgroups of middle-aged children with ADHD (low-motivational vs. high-motivational condition) in a pretest-training-posttest study by means of a computerized WM training (referring to Klingberg et al., 2005). The training task either included or excluded added game elements. At a certain point during testing, the experimenter offered the children to train more sequences before leaving the observation room under false pretenses. Motivation was measured, amongst other factors, as the number of additionally performed sequences during the experimenter's absence time. Training gains were defined as improvements in task performance, while (near) transfer effects were measured by improvements in performance on a new but structurally similar WM task. Results indicated that the high-motivational group chose significantly more voluntary training sequences, pointing to a higher training willingness in the game-setting condition. Moreover, the high-motivational group outperformed the low-motivational one on training performance throughout all practice sessions and showed significant more near transfer. However, one problem of that study was that the motivational score was confounded with the training duration. Choosing additional sequences increased the training experience. Although this confounding was controlled by a statistical *post-hoc* procedure, one further aim of the study was to increase the experimental validity by measuring motivation in terms of training willingness independent of the training duration (for details, see Section Training intervention).

Another critical point of the study by Prins et al. (2011) was that in the high-motivational game condition, the difficulty level was not adjusted to performance (i.e., trials of varying difficulty were presented in a random fashion), whereas in the low-motivational condition, the task difficulty was adaptive on a trial-by-trial basis. The authors argued that in a high-motivational game condition, a trial-by-trial adjustment of the difficulty level would have been contraindicated as it would have probably led to larger individual mood fluctuations (i.e., to more individual frustration and, by this, to more current decrease in motivation). However, the difference in the training procedure across conditions strongly limits the interpretation of findings. In the present study, we thus used the same adaptive feedback algorithm in all training conditions.

Finally, another possible source of motivational influence is the level of cognitive-control demands induced by the training condition. As Ach (1935) described in his classic difficulty law of motivation, individuals are assumed to try harder when they perceive the task difficulty to be higher. More recently, Gendolla (2000) found that more demanding tasks increase subsequent effort mobilization. This is especially reflected by the level of behavioral adjustment (for a summary, see also Van Steenbergen, 2011). Relatedly, Sergeant (2000) addresses the cognitive demands of a given task as a main source of influence onto the “energetic pool” of motivation effort in his cognitive-energetic model (CEM), which was originally developed to account for cognitive impairments in children suffering from ADHD. Specifically, even cognitive control might be affected by the interaction of cognitive demands and motivation (see also Sergeant, 2005). Evidence for this view comes from studies investigating conflict processing with the classical Stroop task. With the occurrence of interference, for example in conflicting Stroop trials, an adaptive increase in selective attention has been found (Egner, 2007; see also Van Steenbergen, 2011). In line with these previous findings and theoretical considerations, we contrasted two levels of cognitive-control demands in the training task (active control/single-task training vs. task-switching training) to examine whether this would differentially affect the motivational outcomes (willingness to effort).

### Aims and predictions

To sum up, we aimed at implementing a task-switching training for middle-aged children into a game environment. We designed this game setting by referring to the essentials of the SDT from Ryan and Deci (2000) and to the self-determinative interpretation of the motivational incitement of video-games according to Ryan et al. (2006). We manipulated the training's frame story, the layout, the type of feedback presentation, and the chances to develop in a game-supporting manner. These instructional design properties were supposed to enhance intrinsic motivation and to induce self-determinative feelings. The primary interest of the study is whether this manipulation interacts with cognitive-control demands of the training situation as well as with the training and transfer gains.

To examine this, we varied the Training Type (single-task vs. task-switching training) and the Training Setting (low vs. high-motivational) separately from each other. Importantly, our motivational manipulation was designed to be independent of the amount of training experience and we presented adaptive feedback in all groups. The specific goals of the study are to investigate whether the type of the training and the training setting would affect (1) the training willingness indicating the intrinsic interest in the task; (2) the task performance (processing speed and accuracy) and the switching performance in the training task; and (3) the transfer of training benefits to an untrained switching task (near transfer) as well as to other task domains, such as inhibitory control and verbal WM (far transfer). On the basis of the reported empirical findings and theoretical considerations we specified a number of a-priori predictions.

Regarding (1) the motivational effects on the training situation we expected, first, a higher and more stable training willingness in groups with a high-motivational setting than in groups with a low-motivational setting (cf. Prins et al., 2011). Second, we hypothesized that the training type could also modulate motivational effects due to the difference in the challenge of task demands: We predicted that higher cognitive demands would stimulate the motivational outcomes, that is, groups with a task-switching training should express higher and more stable interest in the task than groups with a single-task training. Third, we assumed an interaction between the motivational setting and the training type: To examine whether the setting has a greater impact on motivation than the training type, we specified the following *nested* contrasts: In the first contrast, we tested differences between the two *low*-motivational groups, and we predicted that the low-motivational switching group should show higher and more prolonged willingness in performing the training task as compared to the low-motivational single-task group (due to the inciting effect of higher task demands). In the second contrast, we tested differences between the two *high*-motivational groups, and we predicted that the switching-training should boost the positive impact of the motivational setting as compared to the low-demanding single-task training; that is, the high-motivational switching group should show greater and more stable interest in the training task than the high-motivational single-task group. A third nested assumption on the interaction of training setting and type was a direct testing of whether the training setting would have greater impact on motivation than the training type: This should be reflected by higher willingness scores and smaller loss of willingness over time in the high-motivational single-task group compared to the low-motivational switching-group.

Regarding (2) the training effects on cognitive task and switching performance, we first hypothesized a higher task performance (i.e., lower latencies and lower errors) as well as larger performance gains over time in task-switching groups than in groups with a single-task training (cf. Karbach, 2008; Karbach and Kray, 2009). We further assumed that the effects on cognitive performance would also differ between the motivational groups, namely the high-motivational setting condition should facilitate any benefits as compared to the low-motivational setting condition. We third expected again an interaction between the motivational setting and the training type. Again, we specified this prediction along three *nested* contrasts: In the first contrast, we tested motivational differences between the two *single*-*task* groups, and we predicted that the low-motivational single-task group should show smaller and less stable training benefits than the high-motivational single-task group due to the induced different levels of willingness in the task. In the second contrast, we tested motivational differences between the two *task-switching* training groups, and we predicted that the high-motivational setting should expand the type-induced benefits, that is, the high-motivational switching group should show larger and more persistent performance gains than the high-motivational single-task group. In the third contrast, we assumed that the training type would have greater impact on cognitive performance than the training setting: This should be reflected by greater performance improvement over time in the low-motivational switching group compared to the high-motivational single-task group.

On the level of the respective switching costs (i.e., on a more proper indicator of cognitive control), we predicted that the high-motivational task-switching group should outperform the low-motivational task-switching group, again due to different levels of willingness in performing the training task.

Finally (3), we assumed that the predicted training effects on task and cognitive-control performance would propagate toward a structurally similar switching task and may also be present in tasks from other task domains, such as inhibition and WM (cf. Karbach, 2008; Karbach and Kray, 2009).

## Materials and methods

### Participants

The initial sample consisted of 56 middle-aged children between the ages of 8.10 and 11.10 years (*mean age* = 9.63 years, *SD* = 0.81, 50% female). They were recruited from a subject pool from Saarland University. For all children, written informed consent from one of their parents was warranted, in accordance with the protocols approved by the local ethics committee. Children were paid eight Euros per hour for participating in the training study, consisting of six sessions with a total duration of the training of approximately 5 or 6 h. Two children had to be excluded from the analysis because they were either not willing to finish the training or because they differed in their training performance more than three SD from the corresponding group mean. Thus, the effective sample comprised 54 children (*age range* = 8.10–11.10 years, *mean age* = 9.64 years, *SD* = 0.80, 48.1% female). The sample was subdivided into four different training groups which received different training procedures. Table 1 shows the demographics for these subgroups, which did not substantially differ from each other (all *p*s > 0.46). Importantly, the gender ratio was kept constant across groups in order to counterbalance a possible gender asymmetry in predilections for video-game playing. There were further no baseline differences between groups regarding practices or usages of video games or personal preferences for video-game playing (all *p*s > 0.14).

**Table 1:** **Descriptive statistics for the effective sample**.

	**Training group**			
	**Single- LM group**	**Single- HM group**	**Switching-LM group**	**Switching-HM group**
n	13	14	14	13
Gender distribution (female/male)	6/7	7/7	7/7	6/7
Age range (years)	8.25–11.08	8.08–10.83	8.25–10.83	8.17–10.75
Mean age (years)	9.78 (0.84)	9.40 (0.86)	9.59 (0.75)	9.83 (0.78)
Mean RT mixing costs (pre)	348 (184)	305 (178)	298 (144)	306 (96)
Mean DSST (pre)	34.1 (5.4)	36.8 (7.8)	37.2 (8.8)	37.5 (8.3)
Mean Spot-a-Word Test (pre)	11.4 (3.6)	10.9 (2.8)	11.8 (2.6)	11.7 (2.7)

Standard deviations (SD) are provided in parentheses. Single-LM group = single-task training in a low-motivational setting. Single-HM group = single-task training in a high-motivational setting. Switching-LM group = task-switching training in a low-motivational setting. Switching-HM group = task-switching training in a high-motivational setting. To prevent baseline differences between groups, subjects were matched to one of the four training groups based on their age, their pretest performance in task switching (mixing costs), their basic fluid abilities (perceptual speed, Digit Symbol Substitution Test/DSST, Wechsler, 1955) and their crystallized abilities (verbal knowledge, Spot-a-Word Test, Lehrl, 1977).

### Procedure and design

In order to evaluate the impact of the motivational setting on the training and the transfer success of the task-switching training, this study adopted a pretest-training-posttest design including an active control group (single task training). Transfer was defined as the performance gain at posttest relative to the baseline performance at pretest. Pretest and posttest sessions (each taking about 90 min) had similar structures and contents, including baseline measurements of single-task and task-switching performance as well as of the performance on a battery of several other cognitive tasks. The training comprised four sessions (each taking about 30–45 min) of intense practicing of either single tasks A or B separately (single task training groups) or switching between both tasks on every second trial (task switching groups), and each under low and high-motivational setting conditions. The training sessions were separated by, at least, 1 day and they took place once or twice a week. All participants were tested either individually or in pairs by one of five experimenters.

### Apparatus

Computerized tests were presented on two IBM-compatible Dell™ Latitude™ D830- notebooks with an Intel^®^ Pentium III Xeon™ 2.43 GHz processor. Stimuli were presented on a 15.4-inch color display with a screen resolution of 1280 × 800 pixels and a color depth of 16 bit (high-color). The computer experiments for pre- and post-test-assessment were programed via E-Prime^®^ 2.0 Professional (Psychological Software Tools, 2012); the training tasks were programed via PsychoPy™ (Peirce, 2009).

### Pretest and posttest assessment

#### Task switching

For the pre- and post-test measurements of task-switching performance, we adapted the alternating-runs paradigm from Karbach and Kray (2009). In this paradigm, the participants were instructed to perform and switch between tasks A and B. Task A was the “food task” in which participants had to decide whether a presented picture showed a fruit or a vegetable and to press the left or right response key according to a given response scheme. Task B was the “size task” in which children were to decide whether the picture was presented in a small or large size and also to respond with the left or right response key. All stimuli were ambiguous and the same response keys were used for both tasks (i.e., the left key press was correct for task A when the picture was a fruit and for task B when the picture was presented in small size, and the right one was correct for task A when the picture was a vegetable and for task B when the picture was presented in large size). The stimulus set consisted of 16 fruit and 16 vegetable pictures, each of which were given in a small and a large version. All children first worked through two practice blocks in which they performed either task A or B separately. Then, they worked through two mixed practice blocks. Afterwards, children performed 16 experimental blocks, eight single-task and eight mixed-task blocks, each block containing 17 trials. These blocks were presented with the block-order sequence of “single, single, mixed, mixed.” The trial procedure was identical for single- and mixed-task trials. Each trial started with a fixation cross lasting 1000 ms and was followed by a target stimulus. The target remained visible on the screen until the subject responded. The subsequent fixation cross appeared after an inter-trial interval (ITI) of 200 ms. At the end of each trial block, the participants received feedback about their mean reaction time and the achieved number of correct responses.

#### Cognitive test battery

In order to assess far-transfer effects of the task-switching training, we applied a battery of structurally dissimilar tasks which were assumed to measure other facets of cognitive control, such as inhibitory control and WM. Each construct was measured by two indicators to increase the reliability of the measurement. The order of the cognitive tasks was kept constant across participants.

***Inhibitory Control***. Color Stroop (cf. Salthouse and Meinz, 1995): Children were presented with color words (e.g., “blue”) or non-color words (e.g., “book”) written in differently colored ink (red, blue, green, or yellow). They were instructed to respond to the ink of the words by pressing one of four response keys. The word meaning either matched the ink color (congruent trials, e.g., “blue” in blue ink), interfered with it (incongruent trials; e.g., “blue” in red ink), or was completely unrelated to color (neutral trials, e.g., “book”). Stroop interference was defined as the difference in performance between neutral and incongruent trials. Children performed two practice blocks à 12 trials and four test blocks à 24 trials. Each trial started with a fixation cross (700 ms) and was followed by the target. The target was presented until a response was given but not exceeding 2000 ms and was followed by an ITI of 700 ms. After each block, children saw a feedback display showing the mean RT and the percentage of correct responses.

AX-CPT (cf. Servan-Schreiber et al., 1996): Children were presented with consecutive pairs of letters (A, F, G, or S followed by X, C, M, or U). The first letter of each consecutive word-pair was the cue (and more precisely, A = target cue and F, G, or S = distracter cues; the distracter cues together referred to as “B”). The second letter was the respective probe (and more precisely, X = target probe and C, M, or U = distracter probes, the latter distracter probes together referred to as “Y”). In 70% of cases, an A was followed by an X. Such AX trials were declared to be the target pair. The remaining third of trials (i.e., AY, BX, and BY trials) were declared to be distracter pairs. After each probe presentation, subjects were asked to press the right response key if they had identified the target pair and to press the left response key if any other combination of cue and probe stimuli was presented. Since AY and BX distracter trials each overlapped on one element with the target pair (either on cue or on probe), these trial types were assumed to produce interference. AX trials (unambiguous target pair) and BY trials (unambiguous distracter pair), though, should not produce interference. Interference was thus defined as the difference in mean performance between interference (AY- and BX-trials) and non-interference trials (AX- and BY-trials). Participants performed two practice blocks à 10 trials and two test blocks à 50 trials. The cue was presented for 500 ms, followed by an inter-stimulus interval of 1500 ms. Then the probe appeared for 500 ms and was followed by a blank of 800 ms where the participant could press the respective response key. After each trial, the participant saw a feedback display (for 1500 ms) showing the mean RT and the correctness of the response.

***Working Memory***. Digit Span Backward Test (cf. Wechsler, 1981): The experimenter read aloud a series of digits and the children were asked to repeat them in reverse order. The sequences' length (i.e., the number of items) was successively increased (on every third trial by one element) starting with an initial set size of three items (maximum span: 9 digits).

Counting Span Task (cf. Kane et al., 2004): Children were presented with consecutive slides showing varying numbers (1–9) of differently colored (green, blue) geometric shapes (circles, squares) on a gray background. They were instructed to count aloud the dark blue circles per slide (covered background task) and to remember the total number (main task). Children had to work in this manner through one to four further slides (resulting set sizes: 1–5 items) until a retrieval cue appeared on the screen, prompting them to recall and to write down all memorized total numbers in order of presentation.

### Training intervention

The task-switching procedure during training was structurally similar to the one applied at pre- and post-test. However, different stimulus material and different tasks (tasks C and D) were used. Participants were presented with pictures of the game characters called “Watermons” (the stimuli were not labeled as such in the low-motivational condition, see Section A game-based framework for a cognitive-control training in children). Participants were to decide whether the Watermons were either equipped with a net or an energy shield (task C), or with an energy ball or a torpedo (task D, see Figure 2). The trial procedure was equivalent to the one of the transfer tasks A and B. In each training session, the children were asked to work through four (in the first session) and two (in the subsequent sessions) practice blocks and through 24 test blocks each containing 17 trials.

The four matched groups received differential training procedures: In the *single-LM group* (single-task training in a low-motivational setting), children performed tasks C and D in low demanding single-task blocks. There was no additional motivational incentive via game elements. In the *single-HM group* (single-task training in a high-motivational setting), single-task practice was embedded in the stimulating game environment. The *switching-LM group* (task-switching training in a low-motivational setting) trained on mixed-task blocks with high executive-control demands. In this group, however, there were no game elements to support the training motivation. The *switching-HM group* (task-switching training in a high-motivational setting) performed a switching training in the high-motivating game setting.

#### Adaptive threshold

Unlike in the study of Prins et al. (2011), we intended to provide adaptive feedback in both the low- and the high-motivational conditions to tailor the task difficulty to the children's abilities. Prins et al. (2011) assumed that potential motivational fluctuations stem from the adaptivity of the training-task feedback rather than from pure game setting. In order to minimize these fluctuations, our feedback was presented after each whole block and not on a trial-by-trial basis. Each RT block result was further added by a bonus, turning the speed threshold to be slightly more liberal: A positive block feedback was individually challenging but not too difficult to achieve. This bonus enabled a continuous winning chance, ensuring that the child maintained a relatively constant interest level by being protected from too much frustration. The algorithm for the adaptive speed threshold was based on the following mathematic rationale:

“To win a block, the median RT of the current block has to be lower than or equal to the median RT of the preceding block, weighted by the previous performance variability and the tournament status [the tournament status was not explicitly labeled as such in the low- motivational condition]. At the beginning of each session, the tournament status amounts to zero points and is reduced or increased by one point with each slower or faster RT block result. The threshold can be calculated based upon the formula

$${\text{Md}}_{\text{i}}\leq {\text{Md}}_{\text{i }-\;1}+{\text{SD}}_{\text{i }-\;1}(-{\text{T}}_{\text{i }-\;1}+2)$$

where Md_i_ denotes the median RT of all correct responses in block i, and SD_i_ denotes the corresponding standard deviation. T_i_ denotes the tournament status after block i. The minus sign in front of T_i−1_ accounts for the adaptivity of calculation: The higher the number of blocks that are won, the higher is the tournament status but the stricter is the threshold, and vice versa. To account for the fact that RTs do not improve linearly but rather converge to an asymptote, each time a two-point bonus is added.”

#### Motivation index

**Figure 4:**
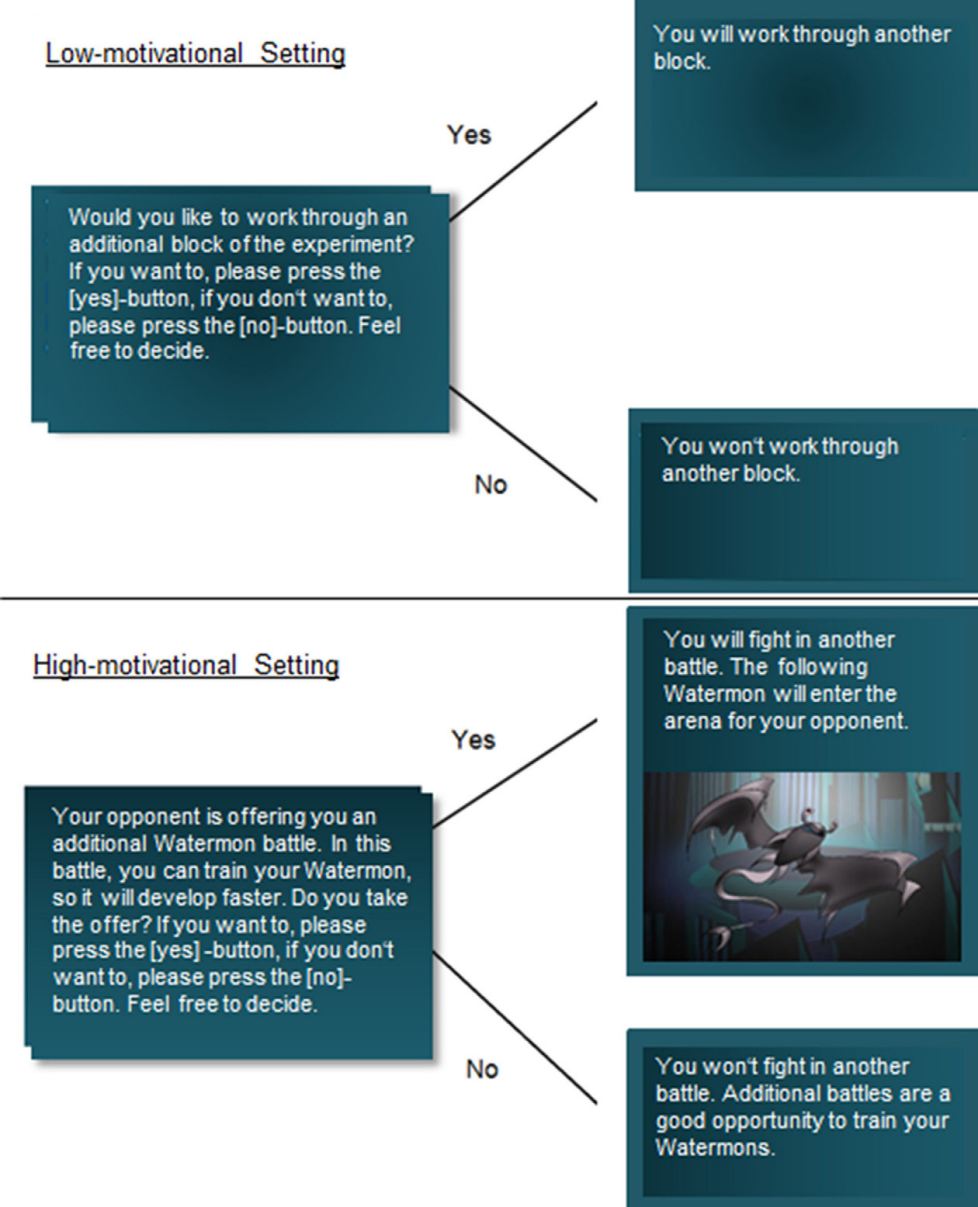
**Different selection windows in respect of the willingness decisions in the low-motivational (upper area) and the high-motivational condition (lower area)**.

In order to obtain an objective index for the children's motivation on the training task and as a manipulation check of the variation in game setting, children of all groups were asked whether they were willing to perform an optional training block (see Figure 4) five times per session. A main goal of the present study was to disentangle the confounding of training motivation and training duration. Therefore, we ensured that children performed 24 experimental blocks *irrespectively* of their actual choice to play additional blocks. This was covered by a pre-programmed algorithm randomizing the positions of the willingness questions during each training session. According to that, the questions appeared variably after each (4 ± 1)^rd/th^ block (but arguably never later than after the 21st one; that is, the last question could never appear accidentally after the very last block when there would be no effective possibility to continue). As a result of the manipulation, children should not become aware of the fact that they did not train any more blocks.

## Results

### Data analysis

Analyses for task switching, Stroop task and AX-CPT were based on median RT for correct responses (ms) and on error rates (%). Practice blocks and the first trial in each block were excluded from data analysis. For task switching, latencies outside the range of 200–3000 ms (training) or to 3500 ms (pre- and post-test) were excluded from analysis (training: 1.83%, pre: 2.80%, post: 1.37%). For Color-Stroop, latencies <250 ms (pre: 1.04%, post: 1.98%), and for AX-CPT, latencies <100 ms were excluded (pre: 0.06%, post: 0.11%). The cut-offs were derived from the empirical RT frequency distributions of each task. Due to a predefined time frame to respond in the Stroop task and the AX-CPT, all trial latencies >2000 ms (Color Stroop) or >1300 ms (AX-CPT) were subsumed under the misses. Error rates were calculated by integrating misses and false alarms. For WM, analyses were based on the number of correct responses.

The findings fall into two parts, each dealing with a separate level of analysis. In the first part, we investigate the benefits of the training on motivation (intrinsic interest) and on training performance. Performance is viewed as a function of the training type and the motivational training setting. In the second part of the analysis, we examine the scope of transfer of the training benefits to the performance in a structurally similar switching task (near transfer) and in structurally dissimilar “executive” tasks from other task domains, such as inhibitory control and verbal WM (far transfer).

For the analysis of the motivational data (effects on intrinsic interest), one child was excluded from analysis due to accidental data loss. For the evaluation of transfer effects, some further cases were excluded either due to technical problems or because the respective task performances were differing more than three SD from the corresponding group mean. The notes in the performance tables (see Tables 3, 4) indicate the sample sizes which effectively entered the separate transfer-task analyses.

To examine the scope of training and transfer effects, we calculated Cohen's (1977) *d* as the group-specific standardized mean difference in performance based on the cost level between beginning and end of training (or between pretest and posttest, respectively; cf. Verhaeghen et al., 1992). All *d*-values were corrected for small sample bias using the Hedges and Olkin's (1985) correction factor (*d*′; see also Karbach and Kray, 2009).

### Training data

#### Training effects on willingness (intrinsic interest)

To investigate the effectiveness of our motivational manipulation, the number of voluntarily chosen blocks was subjected to a Two-Way ANOVA, including the between-subjects factor Training Group (single-LM/single-HM/switching-LM/switching-HM) and the within-subjects factor Training Session (1/2/3/4). In line with our assumptions, we specified a priori two sets of orthogonal contrasts to interpret the group factor with an increased statistical power. Table 2 (contrast variable: motivation, training willingness) provides an overview of the predictions and the respective contrast coefficients. For convenience, all contrast *t*-values were transformed into *F*-values with 1 numerator df.

**Table 2:** **Orthogonal coefficients for a priori group contrasts**.

**Contrast Variable**	**Prediction**		**Orthogonal coefficients for a priori group contrast**			
			**Single- LM group**	**Single- HM group**	**Switching-LM group**	**Switching-HM group**
Motivation *Training Willingness*	**Prediction (1):** (single-HM + switching-HM) > (single-LM + switching-LM)	Set 1 (main)	−1	1	−1	1
	**Prediction (2):** (switching-LM + switching-HM) > (single-LM + single-HM)		−1	−1	1	1
	**Prediction (3):** (single-LM + switching-HM) ≠ (single-HM + switching-LM)		−1	1	1	−1
	**Prediction (3a):** switching-LM > single-LM	Set 2 (nested)	−1	0	1	0
	**Prediction (3b):** switching-HM > single-HM		0	−1	0	1
	**Prediction (3c):** single-HM > switching-LM		0	1	−1	0
Cognition *Performance on Training and Transfer Tasks*	**Prediction (4):** (switching-LM + switching-HM) > (single-LM + single-LM)	Set 1 (main)	−1	−1	1	1
	**Prediction (5):** (single-HM + switching-HM) > (single-LM + switching-LM)		−1	1	−1	1
	**Prediction (6):** (single-LM + switching-HM) ≠ (single-HM + switching-LM)		−1	1	1	−1
	**Prediction (6a):** single-HM > single-LM	Set 2 (nested)	−1	1	0	0
	**Prediction (6b):** switching-HM > switching-LM		0	0	−1	1
	**Prediction (6c):** switching-LM > single-HM		0	−1	1	0

>One-tailed contrast; ≠, Two-tailed contrast.

**Figure 5:**
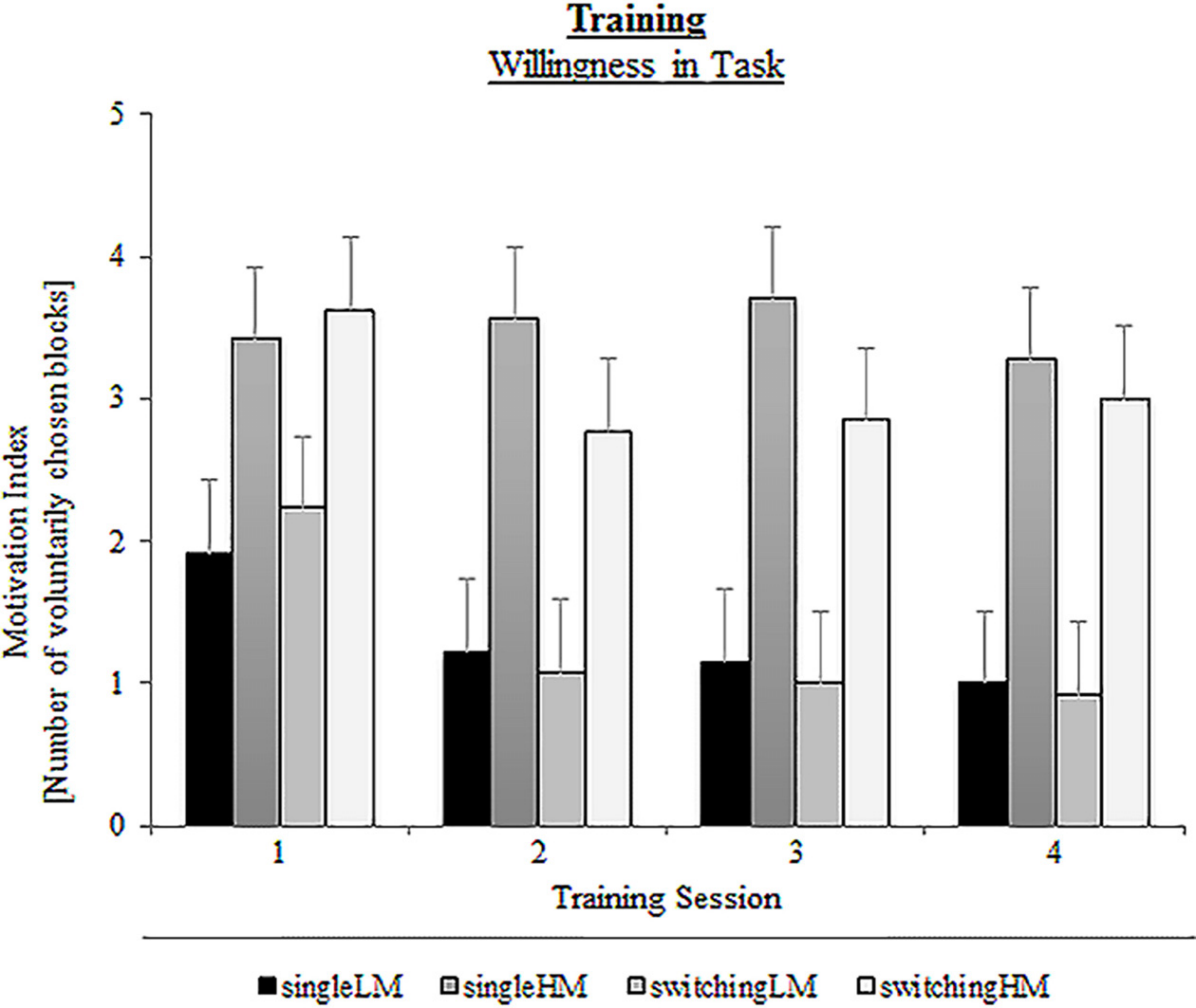
**Motivation index (number of voluntarily chosen blocks) as a function of Training Group (single-LM, single-HM, switching-LM, switching-HM) and Training Session (1,2,3,4)**. Error bars depict standard errors (SE) based on the group x session interaction comparing group conditions of the respective mixed ANOVA according to Jarmasz and Hollands (2009). Note that the selected variance estimators are not suited to compare session conditions.

Our results first revealed an effect on the motivational setting: In Figure 5 it can be seen that the high-motivational (HM) groups showed a higher amount of training interest than the low-motivational (LM) groups, [*F*_(1, 49)_ = 21.81, *p* < 0.001, η^2^_*p*_ = 0.31]. We found no group differences based on training type (*p* = 0.59). This suggests that intrinsic interest did not differ between the single-task training and the task-switching training groups. There were also no specific modulations of the training type within the levels of the training setting (both *p*s > 0.46). However, there was a significant contrast between the single-HM and the switching-LM group, [*F*_(1, 25)_ = 13.91, *p* < 0.001, η^2^_*p*_ = 0.22], indicating that the single-HM group showed a greater willingness to perform additional practice blocks than the task-switching group with a low-motivational setting. Hence, the training setting had a large impact on the training motivation independently of the type of training. We also obtained a main effect for Session, [*F*_(3,147)_ = 5.99, *p* < 0.01, η^2^_*p*_ = 0.11], pointing to a general decrease in the willingness of children to perform additional training blocks over time. However, we found no significant pairwise comparisons on this change (all *p*s > 0.07), indicating that groups did not differ in their decrease of training interest over time.

#### Training effects on task performance

We further compared the training-related improvements in task performance across all groups and ran a Two-Way ANOVA, including the between-subjects factor Training Group (single-LM/single-HM/switching-LM/switching-HM) and the within-subjects factor Training Session (1/2/3/4). We again defined two sets of orthogonal contrasts for the group factor. Table 2 (contrast variable: cognition, performance) summarizes the respective group contrast definitions.

We found that the single-task training groups showed faster latencies than the task-switching groups, [*F*_(1, 50)_ = 5.86, *p* < 0.01, η^2^_*p*_ = 0.10]. We also obtained an effect for the training setting: the groups with an HM-setting generally responded faster than the groups with an LM-setting, [*F*_(1, 50)_ = 10.63, *p* < 0.01, η^2^_*p*_ = 0.17]. Finally, when nesting the training-setting factor beyond the training type, the motivational setting only differed between the switching-training groups, [*F*_(1, 25)_ = 10.18, *p* < 0.01, η^2^_*p*_ = 0.17], and not between the single-task groups (*p* = 0.08). Results revealed a main effect for Training Session, [*F*_(3,150)_ = 21.02, *p* < 0.001, η^2^_*p*_ = 0.30], modulated by the type of training: the groups with a task-switching training showed larger training-related improvements, that is, a faster speeding of responding with increasing practice than the single-task groups, [*F*_(1, 50)_ = 17.39, *p* < 0.001, η^2^_*p*_ = 0.10]. However, the performance change over time was in no way modulated by the setting (all *p*s > 0.29).

**Figure 6:**
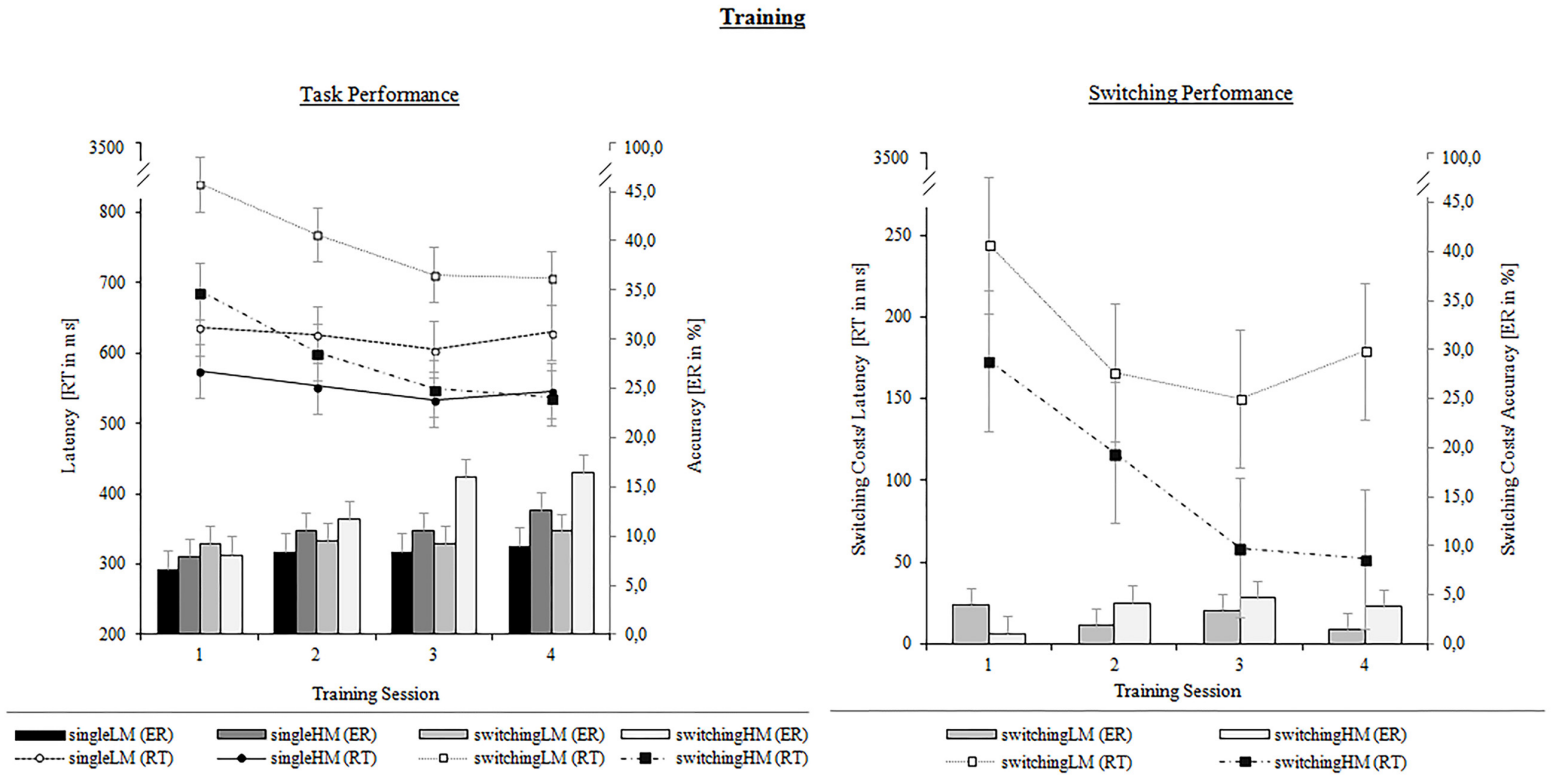
**Training performance on mean latencies (ms) and error rates (%) (left panel) as well as on latency and error switching costs (right panel) as a function of Training Group (single-LM, single-HM, switching-LM, switching-HM) and Training Session (1,2,3,4)**. Error bars depict SE based on the group x session interaction comparing group conditions of the respective mixed ANOVA according to Jarmasz and Hollands (2009). Note that the selected variance estimators are not suited to compare session conditions.

Results on error rates revealed a main effect for Session, [*F*_(3,150)_ = 12.04, *p* < 0.001, η^2^_*p*_ = 0.19], indicating a general decrease of performance accuracy. Regarding the pairwise comparisons, we found neither group main effects nor nested modulations in the expected directions. Nevertheless, there was an unexpected effect for the motivational setting on the change of error rates, [*F*_(1, 50)_ = 6.92, *p* < 0.05, η^2^_*p*_ = 0.04], pointing in the opposite direction: as shown in Figure 6, groups with an HM-setting showed a larger increase of error rates from the first to the fourth training session. These motivational differences were large between the task-switching groups, [*F*_(1, 50)_ = 8.41, *p* < 0.01, η^2^_*p*_ = 0.05], while being unsubstantial between the single-task groups (*p* = 0.42). This result pattern pointed toward a potential speed-accuracy trade-off pronounced in the switching-HM group, which we controlled for in separate analyses (see below).

#### Training effects on switching performance

We further analyzed whether training-related improvements in task switching differed across the two motivational training settings. Data of the two switching groups (switching-LM/switching-HM) were subjected to a Three-Way ANOVA with the between-subjects factor Motivational Setting (low/high), and the within-subjects factors Trial Type (non-switch/switch) and Training Session (1/2/3/4). As we only compared the two task-switching groups to each other, a definition of multiple group comparisons was renounced. In this section, we will focus on switching costs. Thus, we will only report on results including the trial-type factor.

We found reliable switching costs, [*F*_(1, 25)_ = 66.95, *p* < 0.001, η^2^_*p*_ = 0.73], which were modulated by the motivational setting, [*F*_(1, 25)_ = 5.88, *p* < 0.05, η^2^_*p*_ = 0.19]. As can be seen in Figure 6, the switching-training group with an HM-setting showed smaller switching costs than the group with an LM-setting. Switching costs were substantially reduced throughout the practice sessions, [*F*_(3, 75)_ = 5.87, *p* < 0.01, η^2^_*p*_ = 0.19], from 173 ms to 52 ms in the HM-group and from 244 ms to 179 ms in the LM-group. However, training-related changes showed different slopes in the two groups. The switching group with an HM-setting clearly showed a linear decrease in switching costs across the four practice sessions, [*F*_(1, 12)_ = 8.20, *p* < 0.05, η^2^_*p*_ = 0.41], and no empirical support for a quadratic change (*p* = 0.35). The opposite pattern was found for the switching group with an LM-setting, namely a significant quadratic trend, [*F*_(1, 13)_ = 14.57, *p* < 0.01, η^2^_*p*_ = 0.53], but no reliable linear trend (*p* = 0.30). This increase in switching costs between the third and the fourth session was mainly due to a larger improvement on non-switch trials and a stable performance on switch trials in the switching-LM group.

The effect sizes (ES) supported the revealed performance trends: ES for latency switch costs were maximized for the switching-HM group with straight linear gains (*d*′ = 0.92) in comparison to the switching-LM group which showed a rebound at the end (*d*′ = 0.40). Both ES met Klauer's (2001) criterion requiring a minimum size of 0.30 to reach practical relevance.

Results on performance accuracy revealed a main effect for Trial Type, [*F*_(1, 25)_ = 33.08, *p* < 0.001, η^2^_*p*_ = 0.57], pointing to higher error rates on switch than on non-switch trials, and a three-way interaction between Session, Setting and Trial Type, [*F*_(3, 75)_ = 4.80, *p* < 0.01, η^2^_*p*_ = 0.16]. A disentanglement of the latter interaction indicated that the switching-HM group showed an increase in switching error costs from the first to the fourth training session, [*F*_(3, 36)_ = 3.12, *p* < 0.05, η^2^_*p*_ = 0.21], while the LM-group showed a decrease in switching error costs between the first and the fourth session, this being significant at the 5% level, [*F*_(3, 39)_ = 2.92, *p* = 0.05, η^2^_*p*_ = 0.18]. With regard to error switch costs, we found comparable amounts of ES across groups (switching-HM: *d*′ = −0.63; switching-LM: *d*′ = 0.62).

#### Controlling for speed-accuracy trade-offs

Given that the benefit on correct-response speed (or on the latency switch costs, respectively) co-occurred with an increase of errors, especially in the switching-HM group, we assumed group differences in speed-accuracy trade-offs. This assumption might limit the interpretation of the group differences in training and, as a result, in transfer effects. To rule out this possibility of group differences in trade-offs, we correlated latencies and error rates (or the respective switching costs) for the separate experimental conditions. However, these correlations all proved to be negligible (all *p*s > 0.06), with the exception of two significant negative correlations in the single-HM group (*r* = −0.64, and *r* = −0.57, both *p*s < 0.05) and one significant but positive correlation in the switching-HM group (*r* = 0.56, *p* < 0.05).

#### Interim summary of the training data

To sum up so far, analyses of training data first pointed out that our high-motivational game setting was well suited to enhance the willingness to practice, independently of the different demands placed by the training type. The embedding of the switching training into a high-motivational setting also promoted the children's task and switching performance, at least on the latency level. Yet, these improvements on latencies were mitigated by a slight decline of response accuracy.

### Transfer data

#### Analysis of group differences in pretest performance

To account for potential baseline differences between groups, pretest data from all tasks were subjected to ANOVA procedures including the between-subjects factor Training Group (single-LM/single-HM/switching-LM/switching-HM), and, where required, including the respective within-subjects factor Trial Type. The analyses did not display any group main effects or interactions, regarding both latencies and error rates or test scores, respectively. The analyses also did not yield effects on the cost level (all *p*s > 0.11). However, they revealed a different baseline performance between the switching-LM and the switching-HM group on error switch costs, [*F*_(1, 25)_ = 5.01, *p* < 0.05, η^2^_*p*_ = 0.09]. The latter result indicated that any transfer effects concerning error switch costs require cautious interpretation as the transfer effects could simply reflect systematic group differences rather than condition-induced effects.

#### Training effects on near transfer

To compare the near transfer of the training procedure to a structurally similar switching task (see Table 3), data were subjected to a Three-Way ANOVA with the between-subjects factor Training Group (single-LM/single-HM/switching-LM/switching-HM), and the within-subjects factors Trial Type (single/non-switch/switch) and Session (pretest/posttest). Group contrasts were identical to those used for the evaluation of practice effects on task performance. Mixing and switching costs were also defined as orthogonal contrasts by attaching weights to the levels of the Trial-Type factor: Mixing costs were calculated as the difference in mean performance between single-task and mixed-task blocks (contrast: 2 -1 -1); switching costs were calculated as the difference in mean performance between non-switch and switch trials within mixed-task blocks (contrast: 0 −1 1).

**Table 3:** **Performance on the near-transfer task as a function of Training Group (single-LM, single-HM, switching-LM, switching-HM) and Session (pretest, posttest)**.

	**Single-LM group**				**Single-HM group**				**Switching- LM group**				**Switching-HM group**			
	**Pretest**		**Posttest**		**Pretest**		**Posttest**		**Pretest**		**Posttest**		**Pretest**		**Posttest**	
	***M***	***SD***	***M***	***SD***	***M***	***SD***	***M***	***SD***	***M***	***SD***	***M***	***SD***	***M***	***SD***	***M***	***SD***
**LATENCIES (ms)**																
Single trials	830	91	763	164	854	176	745	246	840	202	794	155	878	161	672	152
Nonswitch trials	986	175	803	210	1004	202	814	227	998	216	811	163	1020	144	689	164
Switch trials	1371	275	1064	326	1314	287	1047	389	1279	279	1063	271	1348	255	838	314
**ERROR RATES (%)**																
Single trials	6.3	5.6	9.6	6.8	6.6	5.6	10.4	4.6	7.5	7.2	8.2	7.2	7.3	8.7	12.7	6.2
Nonswitch trials	10.2	7.2	8.2	6.1	9.9	5.9	10.4	6.8	8.8	7.1	7.1	5.9	9.9	7.3	13.5	8.4
Switch trials	11.1	6.7	10.5	7.6	14.1	7.0	16.8	7.0	12.1	8.7	10.2	7.5	9.2	6.7	19.6	11.2

Single-LM group (n = 11); Single-HM group (n = 14); Switching-LM group (n = 14); Switching-HM group (n = 13).

Results on latencies revealed a main effect for Session, [*F*_(1, 48)_ = 69.54, *p* < 0.001, η^2^_*p*_ = 0.59], pointing to a general speeding of performance at posttest. We obtained a significant contrast between the LM- and the HM-condition on this change, [*F*_(1, 48)_ = 3.76, *p* < 0.05, η^2^_*p*_ = 0.08], indicating that the high-motivational condition led to greater improvement on reaction times. The motivational setting only differed between the switching groups, [*F*_(1, 25)_ = 7.56, *p* < 0.01, η^2^_*p*_ = 0.14], reflecting the bounded protrusion of the switching-HM group. In addition, results indicated reliable mixing costs, [*F*_(1, 51)_ = 179.34, *p* < 0.001, η^2^_*p*_ = 0.78], as well as switching costs, [*F*_(1, 51)_ = 142.01, *p* < 0.001, η^2^_*p*_ = 0.74]. Both mixing and switching costs were substantially reduced from pretest to posttest, [*F*_(1, 51)_ = 58.68, *p* < 0.001, η^2^_*p*_ = 0.54], and [*F*_(1, 51)_ = 14.30, *p* < 0.001, η^2^_*p*_ = 0.22], respectively. Most importantly for the present study, we obtained a significant contrast on the change of switching costs between the switching-HM group and the switching-LM group, [*F*_(1, 48)_ = 4.41, *p* < 0.05, η^2^_*p*_ = 0.09]. This emphasizes that a combination of a switching training and a game setting led to a stressed gain in task-set shifting. However, the change of mixing costs was neither modulated by the training type (*p* = 0.63) nor by the motivational setting (*p* = 0.49).

Considering the ES for near transfer, we found the largest ES in the switching-HM group for the RT switch costs (*d*′ = 0.86) as compared to the other groups (*d*′ = 0.15–*d*′ = 0.57). In contrast to variance-analytical results, we also found the ES for the RT mixing costs in the switching-HM group (*d*′ = 1.68) appearing to be distinctly larger than the ES in the other groups (*d*′ = 0.64–*d*′ = 1.12).

**Figure 7:**
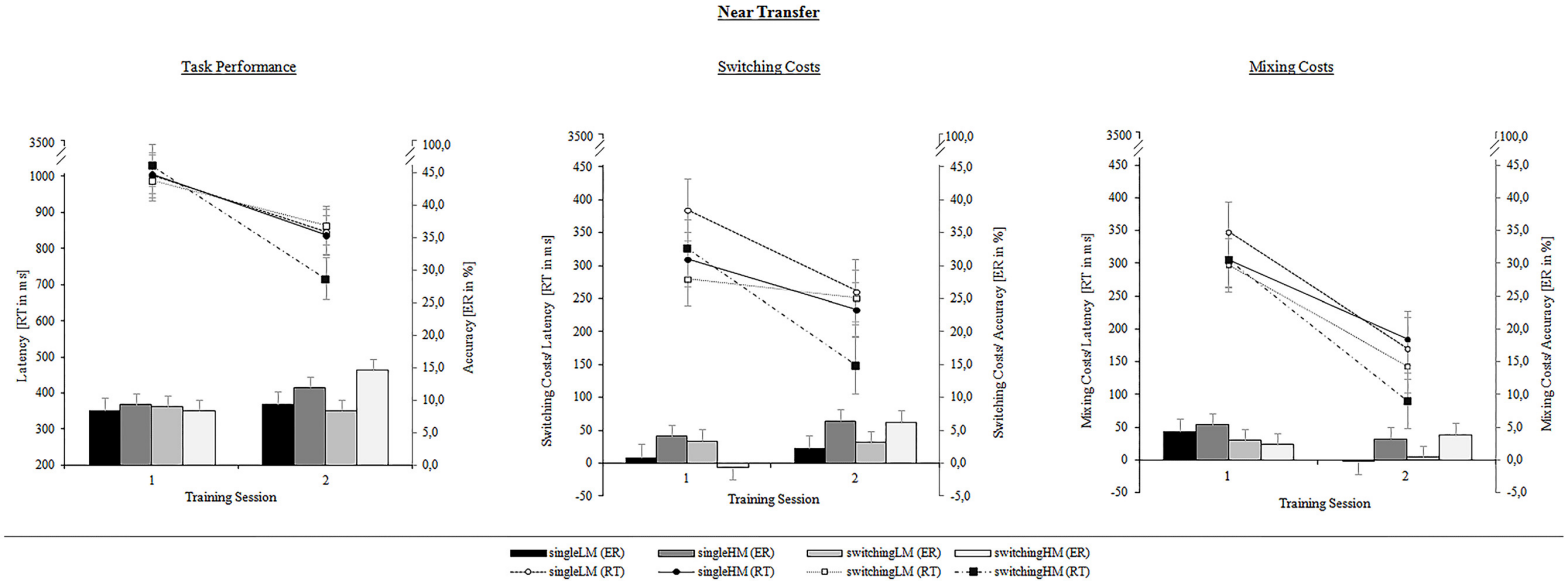
**Near-transfer performance on mean latencies (ms) and error rates (%) (left panel) as well as on the respective switching costs (middle panel) and mixing costs (right panel) as a function of Training Group (single-LM, single-HM, switching-LM, switching-HM) and Session (pretest, posttest)**. Error bars depict SE based on the group x session interaction comparing group conditions of the respective mixed ANOVA according to Jarmasz and Hollands (2009). Note that the selected variance estimators are not suited to compare session conditions.

On the level of accuracy, we yielded a main effect for Session, [*F*_(1, 48)_ = 4.60, *p* < 0.05, η^2^_*p*_ = 0.09], indicating an increase in errors from pre- to post-test. This increase was again more pronounced for the HM-groups than for the LM-groups, [*F*_(1, 48)_ = 6.50, *p* < 0.05, η^2^_*p*_ = 0.13]. Results also revealed reliable mixing costs, [*F*_(1, 51)_ = 18.18, *p* < 0.001, η^2^_*p*_ = 0.26], and switching costs, [*F*_(1, 51)_ = 47.36, *p* < 0.001, η^2^_*p*_ = 0.48]. Mixing costs were slightly reduced (but not in the switching-HM group, see Figure 7), [*F*_(1, 51)_ = 2.96, *p* < 0.05, η^2^_*p*_ = 0.05]. Switching costs increased from pre- to post-test, [*F*_(1, 51)_ = 5.61, *p* < 0.05, η^2^_*p*_ = 0.10]. This specific increase of switching costs was larger for the switching-HM group than for the switching-LM group, [*F*_(1, 25)_ = 5.95, *p* < 0.05, η^2^_*p*_ = 0.12]. However, the latter group difference needs to be interpreted under consideration of the revealed baseline differences in error switch costs. The ES for error costs were, in essence, congruent with the variance-analytical results. That is to say, the switching-HM group yielded maximum ES for the increase in error switching costs (switching-HM: *d*′ = −1.46) as compared to the other groups (single-LM: *d*′ = −0.27; single-HM: *d*′ = −0.48; and especially as compared to the switching-LM group: *d*′ = 0.05), while ES for mixing costs showed similar values across groups, ranging from *d*′ = −0.23 to *d*′ = 0.37; only the single-LM group revealed pronounced benefits for ER costs with an ES of *d*′ = 0.91.

#### Controlling for speed-accuracy trade-offs

We controlled for potential group differences in speed-accuracy trade-offs; group correlations, however, were again negligible or rather positive (all *p*s > 0.10, with the exception of one reliable positive correlation in the single-HM group for non-switch trials at posttest, *r* = 0.62, *p* < 0.05).

#### Interim summary of the near-transfer data

To sum up, the training benefits projected fairly consistently onto latencies and specific switch costs in the near-transfer task: a task-switching training embedded into a high-motivational setting led to the highest gain in response speed and to the largest reduction of switching costs. The latter condition also showed a larger increase of error rates, which again proved to be unsubstantial. Statistically, we found no differential effects on global mixing costs even though the ES pointed to such an advantage for the high-motivational switching condition.

#### Training effects on far transfer

**Figure 8:**
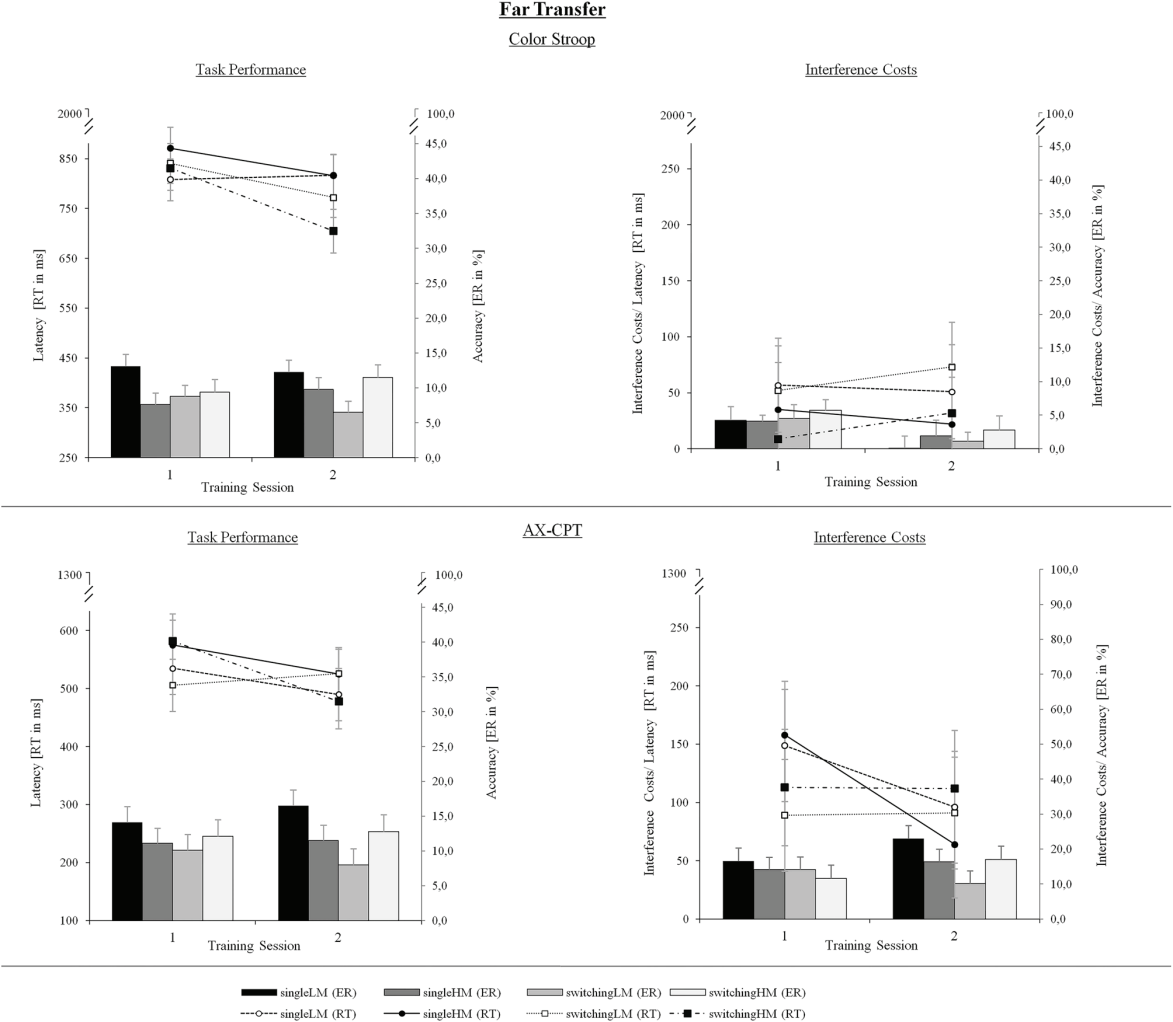
**Far-transfer performance on inhibitory control: mean latencies (ms) and error rates (%) (upper left panel) as well as respective interference costs (upper right panel) of the Stroop task; mean latencies (ms) and error rates (%) (lower left panel) as well as respective interference costs (lower right panel) of the AX-CPT, as a function of Training Group (single-LM, single-HM, switching-LM, switching-HM) and Session (pretest/posttest)**. Error bars depict SE based on the group x session interactions comparing group conditions of the respective mixed ANOVAs according to Jarmasz and Hollands (2009). Note that the selected variance estimators are not suited to compare session conditions.

*Inhibition.* With regard to inhibitory control, we analyzed the Stroop task as well as the AX-CPT (see Figure 8). In both cases, data were subjected to a Three-Way ANOVA with the between-subjects factor Group (single-LM/single-HM/switching-LM/switching-HM), and the within-subjects factors Trial Type (Stroop: neutral/congruent/incongruent, and AX-CPT: AX/AY/BX/BY) and Session (pretest/posttest). We used the same group contrasts as in the previous section. Interference costs were defined as contrasts of the respective trial-type levels: For the Stroop task, interference was calculated as the difference in mean performance between neutral and incongruent trials (contrast: 1 0 −1), and for the AX-CPT as the difference between non-interference trials (AX,BY) and interference trials (AY,BX; contrast: 1 −1 −1 1).

*Color Stroop.* We obtained a significant main effect for Session, [*F*_(1, 48)_ = 21.27, *p* < 0.001, η^2^_*p*_ = 0.31]. This effect was dependent on the training type, [*F*_(1, 48)_ = 7.45, *p* < 0.01, η^2^_*p*_ = 0.14], and on the motivational setting, [*F*_(1, 48)_ = 5.76, *p* < 0.05, η^2^_*p*_ = 0.11], showing that both factors contributed to a reduction of latencies. We obtained reliable interference costs, [*F*_(1, 51)_ = 70.11, *p* < 0.001, η^2^_*p*_ = 0.58], which did not change over time (*p* = 0.54) and were not modulated by group (all *p*s > 0.07). Accordingly, ES for latency interference costs were low and comparable across groups (*d*′ = 0.07–*d*′ = −0.32). With regard to accuracy, the analysis revealed an increase of errors from pre- to post-test, [*F*_(1, 48)_ = 13.98, *p* < 0.001, η^2^_*p*_ = 0.23]. We also found interference costs as neutral trials were substantially better performed than incongruent trials, [*F*_(1, 51)_ = 19.69, *p* < 0.001, η^2^_*p*_ = 0.28], and those costs were significantly reduced from pre-to post-test, [*F*_(1, 51)_ = 7.53, *p* < 0.01, η^2^_*p*_ = 0.13]. Nevertheless, on errors and their respective costs, there were no group-differential modulations (all *p*s > 0.09; the amounts of ES being comparable across groups, ranging from *d*′ = 0.32 to *d*′ = 0.57).

*AX-CPT.* We found a main effect for Session on response latencies, [*F*_(1, 48)_ = 6.51, *p* < 0.05, η^2^_*p*_ = 0.12], pointing to a speeding from pre- to post-test throughout all groups. This improvement was again more pronounced in the HM- than in the LM-groups, [*F*_(1, 48)_ = 3.50, *p* < 0.05, η^2^_*p*_ = 0.07]. The motivational setting differed only between the switching groups, [*F*_(1, 48)_ = 6.15, *p* < 0.01, η^2^_*p*_ = 0.12], and not between the single-task groups (*p* = 0.91). More importantly, we found reliable interference costs, [*F*_(1, 51)_ = 120.34, *p* < 0.001, η^2^_*p*_ = 0.73], reflected by a better performance in non-interference trials compared to interference trials. Interference costs were substantially reduced over time, [*F*_(1, 51)_ = 8.82, *p* < 0.05, η^2^_*p*_ = 0.17]. However, against our assumptions, this reduction was larger for the single-task groups than for the task-switching groups, [*F*_(1, 48)_ = 9.42, *p* < 0.01, η^2^_*p*_ = 0.18]. The latter result was supported by the pretest-posttest ES, which were larger after single-task (*d*′ = 0.81–*d*′ = 0.97) than after switching training (*d*′ = −0.02–*d*′ = 0.01).

The error rates did not change over time (*p* = 0.75). We found reliable interference costs, [*F*_(1, 51)_ = 115.31, *p* < 0.001, η^2^_*p*_ = 0.69], indicating larger errors for interference trials compared to non-interference trials. Interference costs were, however, not modulated by group (all *p*s > 0.08). The ES for error interference costs pointed to a slightly greater increase in the switching-HM group (*d*′ = −0.49) as compared to the other groups (single-LM: *d*′ = −0.38; single-HM: *d*′ = −0.14; switching-LM: *d*′ = 0.32).

*Working memory*. We finally ran analyses on the WM domain (see Table 4). Both for the Digit Span Backward Test as well as the Counting Span Task, data were subjected to a Two-Way ANOVA with the between-subjects factor Training Group (single-LM/single-HM/switching-LM/switching-HM) and the within-subjects factor Session (pretest/posttest). The group factor was interpreted based on the same contrasts as in the previous section. For the Digit Span Backward Test, we obtained a significant main effect for Session, [*F*_(1, 50)_ = 5.65, *p* < 0.05, η^2^_*p*_ = 0.10], pointing to an increase in the number of correct responses from pre- to post-test. However, we found no significant group-differential transfer effects on WM, nor for the Digit Span Backward Test (all *p*s > 0.27) nor for the Counting Span Task (all *p*s > 0.50). In line with these findings, the ES were quite low for both measures (in the Digit Span Backward Test ranging from *d*′ = 0.06 to *d*′ = 0.32, with a slightly higher ES of *d*′ = 0.56 in the switching-LM group; and in the Counting Span Task ranging from *d*′ = -0.06 to *d*′ = 0.18).

**Table 4:** **Performance on far-transfer tasks as a function of Training Group (single-LM, single-HM, switching-LM, switching-HM) and Session (pretest, posttest)**.

	**Single-LM group**				**Single-HM group**				**Switching- LM group**				**Switching-HM group**			
	**Pretest**		**Posttest**		**Pretest**		**Posttest**		**Pretest**		**Posttest**		**Pretest**		**Posttest**	
	***M***	***SD***	***M***	***SD***	***M***	***SD***	***M***	***SD***	***M***	***SD***	***M***	***SD***	***M***	***SD***	***M***	***SD***
**STROOP TASK (INHIBITORY CONTROL)^a^**																
**Latencies (ms)**																
Neutral trials	802	116	816	187	877	185	827	182	832	132	752	127	835	178	705	115
Incongruent trials	859	116	867	212	912	186	848	223	883	145	826	172	843	173	737	158
**Error rates (%)**																
Neutral trials	6.5	7.8	13.1	7.0	3.6	6.1	9.6	7.9	4.2	5.6	6.0	5.0	3.6	4.2	9.7	9.2
Incongruent trials	10.8	12.1	13.1	9.1	7.7	7.4	11.5	6.6	8.7	8.1	7.1	4.5	9.4	5.7	12.5	7.3
**AX-CPT (INHIBITORY CONTROL)^b^**																
**Latencies (ms)**																
AX	463	183	442	143	495	88	515	169	457	127	497	157	546	172	453	165
AY	736	150	653	117	773	114	692	178	695	148	678	166	755	173	632	145
BX	483	267	424	166	535	202	422	152	406	219	465	254	522	251	435	217
BY	457	211	443	169	489	142	471	147	464	234	464	269	506	206	390	210
**Error rates (%)**																
AX	3.9	4.3	5.3	4.3	3.5	2.7	3.6	3.0	2.7	3.6	3.6	2.3	4.3	3.5	3.4	3.6
AY	34.6	29.9	47.2	33.1	25.1	18.2	25.1	15.0	28.5	19.1	21.5	24.2	23.3	13.0	31.9	20.4
BX	10.1	11.6	8.8	12.6	11.2	11.1	14.3	18.7	5.9	9.6	4.6	7.8	12.6	11.4	10.6	14.5
BY	7.7	15.4	4.7	6.7	4.5	6.7	2.9	6.1	3.4	5.4	2.3	4.4	8.3	11.9	5.3	10.4
**DIGIT BACKWARD SPAN (WORKING MEMORY)^c^**																
**Number correct**																
	4.9	1.8	5.5	2.0	4.6	2.0	5.3	2.2	5.1	2.1	6.4	2.2	5.1	1.4	5.2	1.7
**COUNTING SPAN (WORKING MEMORY)^d^**																
**Number correct**																
	3.5	1.3	3.7	1.5	3.6	1.6	3.5	1.9	3.1	1.2	3.2	1.3	3.5	1.4	3.8	1.8

a Stroop Task: single-LM group (n = 13); single-HM group (n = 13); switching-LM group (n = 14); switching-HM group (n = 12).

b AX-CPT: single-LM group (n = 13); single-HM group (n = 14); switching-LM group (n = 13); switching-HM group (n = 12).

c Digit Backward Span: single-LM group (n = 13); single-HM group (n = 14); switching-LM group (n = 13); switching-HM group (n = 13).

d Counting Span: single-LM group (n = 13); single-HM group (n = 14); switching-LM group (n = 13); switching-HM group (n = 13).

#### Interim summary of the far-transfer data

By relating the results of far transfer to the ones of training or near transfer, we were able to demonstrate that the latency changes in the inhibition tasks partly reflected the former findings: For both measures (Stroop task, AX-CPT), the switching-HM group entered the highest profit on the response dynamics. However, this distinct benefit of the switching-HM group did not extend to the level of interference costs. On this level, the training failed to consistently differ between groups (Stroop task). For the AX-CPT, however, the training differed as the single-task groups showed the largest reduction of interference costs. There was again a slight decline of accuracy (at least in the Stroop task), which was not as distinctive as in the tasks of training or near transfer. On WM we found no group-differential modulations.

## Discussion

The present study aimed at determining the impact of a motivational setting on the training and the transfer success of a cognitive-control intervention in middle-aged children. To address this issue, we created a game version of a task-switching training based on the principles of the SDT (Ryan et al., 2006). We contrasted the game's effects on intrinsic interest and cognitive performance in a task-switching training (against a single-task control condition) with a low-motivational, standard setting. Importantly, our motivation score was independent of the amount of training experience as we ensured that the willingness decisions had no actual effects on the further training course. The adaptivity of the training procedure (here by providing adaptive feedback) was also kept constant across groups.

### Training effects on willingness (intrinsic interest)

With regard to the motivational outcomes, the results of this study first indicated that the training setting with game elements yielded a greater willingness of the children to additionally practice the task. This finding validates the efficiency of our motivational manipulation and underlines the value of an implementation of the SDT to foster training motivation in children (cf. Ryan et al., 2006). We hypothesized that this interest in the training task would be also modulated by the training type (single-task vs. task-switching training), providing differently challenging task demands for the children. However, the results of the present study did not support such an interaction. This finding might even provide a clearer insight into the influence of motivation on performance outcomes, which, in turn, can be interpreted as being purely caused by the setting. Results also revealed that the training motivation, here the willingness to perform additional task blocks (reflecting the intrinsic interest in the training task), clearly declined with an increasing amount of practice. This probably reflects a fatigue effect which frequently co-occurs with a test-retest setting (e.g., Mitchell and Jolley, 2009). The reduction in training motivation was also not softened by the high-motivational setting as the interest declined independently of the reinforcement by game elements. Hence, the present study provided an important insight: although a game setting generally led to a greater training willingness, this pure setting was not sufficient to overcome the motivational loss over time in children. Perhaps, our proposed framework for operationalizing the interplay between motivation and cognitive control (cf. Figure 1) did not fully account for the moderation role of the broad spectrum of pre-existent motivational trait differences. In the future, our intrinsic variables should further be systematically balanced against extrinsic incentives, such as the magnitude of material incentives (see also Dovis et al., 2011; Kleinsorge and Rinkenauer, 2012), in order to provide the more persistent drive to motivation. As a result, an important extension of our framework for future studies may be both to cover a wider range of pre-existent individual differences in motivation (at the trait level, i.e., other *personal* variables) as well as to consider other extrinsic rewards (i.e., other *environmental* variables) in order to examine if this revised approach would be more suitable to avoid a motivational loss in children as the time spent on the task increases.

### Training effects on task and switching performance

Regarding training benefits on latencies, we found the highest gain in the combined switching-HM group throughout all training sessions. This fully concurs with our hypothesis: the induced training interest will mobilize training willingness and lead to higher levels of task engagement. Regarding switching performance, both motivational groups showed a decrease of switching costs over time. However, the switching-LM group revealed a rebound in the fourth training session: A comparison of RTs indicated that the latter effect was driven by a linear decrease in non-switch trials and a stagnating performance in switch trials from the third to the fourth training session. In contrast, we found an additional benefit of the high-motivational setting, which is reflected by a straight linear decrease of costs in the switching-HM group (without a stagnation of performance on switch-trials against the end of the training). Thus, the HM-setting might have canalized an onward improvement of control processes that were necessary to switch between tasks.

The switching-HM group showed better performance compared to the respective LM-group right from the beginning of the training although both groups did not differ in task-switching performance at pretest serving as a baseline measure. Thus, our HM-setting seemed to have exerted an immediately positive impact on task and switching performance, suggesting that game elements are well suited to enhance task engagement and cognitive-control performance on the top of training interest (training willingness).

However, a major point for discussion was the concurrent increase of errors and error costs over time. This increase also extended to the transfer measurements and was most pronounced in the switching-HM group. This finding pointed to a shift from accurate to faster task execution, particularly in the latter condition. We found no direct statistical support for the assumption of group differences in speed-accuracy trade-offs. If any, we revealed two negative speed-accuracy correlations to be substantial for the single-HM group (and not for the switching-HM group deemed to be the most critical one) on the training task, which yet turned out to be positive on the near-transfer task. However, the variance-analytical results did point to the presence of speed-accuracy trade-offs, at least in terms of a general trend. Davidson et al. (2006) describe a general developmental change course of speed-accuracy trade-offs in executive tasks with an increased impulsiveness at younger age. Relatedly, Karbach (2008) revealed a progressive response behavior at the expense of accuracy for the children group in her task-switching study. Similarly, our findings might thus be interpreted as reflecting the developmental trajectory of trade-offs, meaning that children, in general, do not take the time necessary for precision. This response imbalance should, however, not be critical for the interpretation of group modulations on performance.

To interpret the group-differential effects in our study consistently, we prioritized the latency measures, similar to many previous training studies (e.g., Karbach and Kray, 2009; Zinke et al., 2012). Some studies consider the percentage of correct responses to be the more sensitive measure in childhood (due to the higher variability on reaction times in children, e.g., Davidson et al., 2006). In our specific case, latencies might be the more valid criterion for children as well. We aimed at examining children's motivation, which is, by definition, an energizing force (Sergeant, 2000; Locke and Braver, 2010) that acts in a driven manner and may therefore preferentially affect response dynamics and the speed of processing. In support of this, previous research on commercial video-game playing revealed pronounced benefits on processing speed (e.g., Dye et al., 2009).

### Training effects on near and far transfer

With regard to near transfer, we found that the switching-HM group showed an advantage not only in reducing response latencies but also in reducing latency switch costs as compared to the respective LM group. This influence of the motivational setting, though, was limited to the switch costs, that is, to switching at trial-to-trial transitions. The motivational influence thus specifically affected the proper abilities of reconfiguring task-sets. We found no group-differential effects on mixing costs, which means that children uniformly improved their ability to globally master the task-switching situation (i.e., to maintain and select the two task-set rules). Interestingly, there seems to be a critical age of 11 years (which was, notably, the upper limit of our sample's age range), which may dissociate the genuine developmental trajectory of the ability to switch back and forth between two task rules from the developmental curve of the ability to select and to maintain different rules (Huizinga and van der Molen, 2007; see also Karbach and Unger, 2014): Huizinga and van der Molen (2007) showed that the former shifting abilities (which might be reflected in the amount of the switching costs in the task-switching paradigm) critically approach the adult performance by the age of 11 years. Yet, the rule maintenance abilities (which might be reflected in the amount of global mixing costs) reach adult levels only by the age of 15 years. In the present study, we had assumed an interplay between motivational and cognitive components. Critically, our motivational manipulation in children, ranging from 8–11 years, might have crossed exactly the steep genuine developmental shift in switching abilities. That is to say, our motivational impetus might have tapped into the natural maturation process of the shifting system. However, this acceleration effect might have not been tracked on mixing costs as maintenance abilities continue to develop more slowly into adolescence. On the level of mixing costs, the highest mutual synergies between motivation and cognition would then be expected later than by the age of 11. Nonetheless, the ES did hint at an upcoming (but just slowly unfolding) advantage for the switching-HM group regarding the reduction of mixing costs.

Noteworthy, the single-task groups also showed reductions in switching and mixing costs, maybe indicating a mere practice gain driven by task familiarization. As Kray et al. (2012) proposed, such medium transfer effects after single-task training can be due to ambiguous stimuli in a single-task condition. This may result in a certain amount of inhibitory-control training even with low demands.

Regarding far transfer to inhibitory control, we only found improvements for the switching-HM group in terms of faster responding in the Stroop task and the AX-CPT. However, we revealed no change of interference costs for the Stroop task. With regard to the descriptive pattern, we could state that, for the present study, the general ratio of costs was very low right from the beginning. Interference costs were around 50 ms at pretest, leaving little room for further improvement. Davidson et al. (2006) emphasized that the true demanding cognitive requirement is imposed by the switching context, that is, by a continuous claim for switching back and forth between tasks. The classic Stroop task, however, is virtually organized in a single-task setting and thus lacks the most demanding condition: to switch between responding to the ink of the words once (as in the present case) and responding to the meaning of the words once, respectively. An implementation of the Stroop task in a mixed-task procedure, which might be more tailored to the difficulty level of the task-switching training task, could have prevented the ceiling effects at pretest. The lack of transfer to inhibitory control stood in sharp contrast to the study of Kray et al. (2012), conducted on children with ADHD. This might imply that typically developed middle-aged children may not have as many impairments in classic Stroop interference as clinical subgroups suffering from ADHD. Those subgroups might have more need for compensation. Karbach and Kray (2009), however, did find far transfer to Stroop inhibition in healthy participants as well. However, they employed two various Stroop versions with different stimulus dimensions (i.e., a Color Stroop version and a Number Stroop version) and collapsed data across both variants. Differences in the stimulus complexity between both measures may have reduced the advent of ceiling performances in children at the beginning of the training.

Against our assumptions, for the AX-CPT, we found a change of interference costs pointing to a larger advantage for the single-task groups. Hussey and Novick (2012) emphasize that transfer highly depends on whether the involved (training and transfer) tasks provoke similar processing demands through a shared task structure. Against this background, our findings would indicate that the task structure (or the tapped processing demands, respectively) of the AX-CPT task, which was employed here, resembled the one of the single-training task and not the one of the switching-training task. This might have been expected since the single task required the participant's vigilance to respond to a continuous low-demanding stimulus stream; and this is similarly necessary to meet the demands of the AX-CPT in the present variant. Therefore, the latter tasks might have shared higher proportions of variance in their task structures, providing more common ground for transfer effects. To our best knowledge, the AX-CPT has not yet been applied as a transfer measure of inhibitory control in previous task-switching training studies. We therefore suggest that this result is a task-specific one that should be thoroughly addressed in future research.

Finally, we did not find any transfer to WM, which was again in contrast to our expectations formed by the results of Karbach and Kray (2009). However, the researchers prorated a composite score for both a verbal as well as a visuo-spatial WM span measure, while, in the present study, we assessed transfer benefits solely by means of verbal WM measures. Visuo-spatial facets of WM were possibly more sensitive to get in resonance with the trained abilities; alternatively, only the aggregation across different modalities of WM resulted in a sufficient amount of task power to uncover the latent transfer to this cognitive domain. Karbach and Kray (2009) even stated that their far-transfer results should be interpreted with caution as such effects might be hard to replicate in small samples. They could themselves only reveal transfer benefit on the WM domain if they collapsed data across three different task-switching groups (it should be noted that the researchers also varied the usage of verbal self-instruction and the variability of the training task between the task-switching groups, each separate group comprising only 14 participants). Only the aggregation across the different types of the treatment resulted in an analyzable sample size of *n* = 42 participants, providing sufficient power to detect transfer on WM. Furthermore, other studies also failed to replicate the large transfer effects of Karbach and Kray (2009) even though they adopted very similar training procedures (but administered to adult age brackets; e.g., Von Bastian and Oberauer, 2013; Pereg et al., 2013).

The results on near and far transfer effects of this study are only partly in line with previous findings: we revealed rather limited transfer of a switching training, and even if this was embedded in a high-motivational game setting. This lack of clear benefits, which have previously been found (e.g., Kray et al., 2012), may also be due to differences in the susceptibility for motivational input between typically developed children and subclinical groups of children with ADHD. Children with ADHD show a genuine motivation deficit (e.g., Haenlein and Caul, 1987) or, in terms of the CEM, a non-optimal energetic state (Sergeant, 2000). According to the compensation view, they could thus gain even more from a motivational manipulation, which is supported by the findings of Dovis et al. (2011). The authors examined the motivational sensitivity of children with ADHD to different reinforcers, also including a game setting. By comparison of separate benefits from a motivational WM training between children suffering from ADHD and typically developed controls, they found that only children with ADHD gained from a game setting in their persistence of performance while their healthy counterparts rather stagnated. This result promotes the expectation of higher benefits from a combined motivation and cognition training in children with ADHD (see also Sonuga-Barke, 2002; Bioulac et al., 2014), particularly in respect of buffering against fatigue over time.

### Limitations

The present study faces several limitations including the following ones: The sample sizes in the separate groups were small and need to be enlarged in future studies to increase the experimental power. Future research should also warrant a greater transparency of baseline scores and, particularly, of the basic susceptibility of motivational input. This may also include a stronger control of pre-existent individual differences in *broad* motivational tendencies, besides the induced *specific* interest in the training-task. Critically, future research should strive for employing supplemental self-report measures to assess the success of inducing intrinsic interest more thoroughly. Such a multi-method approach, comprising both subjective and objective instruments, would allow a twofold validation of the motivational manipulation aimed at affecting values of self-identity. Moreover, our study did not state which one of the varied game elements (story framework, stimulus material, task labels, goal instructions, feedback presentation, feedback rewards) specifically contributed to the enhancement of interest (or performance). Thus, an important aspect for further studies might be to disentangle the design properties of the motivational setting to determine which elements could energize the most. Finally, it is important to note that the transfer measures in the present study were presented in standard, low-motivational settings. This might have counteracted our motivational modulation on transfer by opening an artificial gap between the effects of the high-motivational training task and the low-motivational transfer tasks.

### Concluding remarks

The results of the present study make an important contribution to existing research on moderating effects on cognitive-control interventions in middle childhood. Motivation (i.e., the incitement provided by the learning environment) seems to explain a considerable proportion of variance in training interest and willingness. The cognitive demands and the difficulty of the task might be less important for training motivation. The motivational impetus by contextual enrichment tapped into cognitive task performance where this effect sustained beyond the training phase and projected, at least partly, onto the effort spent on other tasks (even if those were presented in standard, low-motivational settings). This consistent motivational benefit was limited to the behavioral dynamics (speed of processing) whereas effects on broad cognitive-control processes were rather limited.

We provided a conceptual and operational framework to dissociate the effects of intrinsic interest on hot motivation and on cold cognition. By referring to this hypothesized interaction framework, we can conclude that adding game elements and inducing self-determinative feelings did indeed synergize with task interest but tapped the level of cognitive control only in passing. It might therefore be useful to refine these connections between motivation and cognition by expanding our framework by individual traits. Genuinely inspired from the compensation account, our proposed training program should be applied to subclinical samples with motivational deficiencies.

Synoptically, heeding the framework's basic principles should inspire future research and help to guide the quest for successful motivational design principles for cognitive interventions in childhood.

## Author contributions

Sandra Dörrenbächer, Philipp Matthias Müller, Johannes Tröger and Jutta Kray conceived the research design of the study. Sandra Dörrenbächer carried out the statistical analyses and interpretations and mainly contributed to the writing of the paper. Jutta Kray was the first coordinator, provided substantial support to proofread the manuscript and gave the final approval of the version to be published. Philipp Matthias Müller and Johannes Tröger suggested the initial research idea to examine the task-switching training in a game-based environment and created and implemented the game setting. Intellectual content was collectively revised by all authors. All authors read and approved the final manuscript.

### Conflict of interest statement

The authors declare that the research was conducted in the absence of any commercial or financial relationships that could be construed as a potential conflict of interest.
